# Time‐varying measures of cerebral network centrality correlate with visual saliency during movie watching

**DOI:** 10.1002/brb3.2334

**Published:** 2021-08-26

**Authors:** Akitoshi Ogawa

**Affiliations:** ^1^ Faculty of Medicine Juntendo University Bunkyo‐ku Tokyo Japan; ^2^ Brain Science Institute Tamagawa University Machida Tokyo Japan

**Keywords:** attention network, functional magnetic resonance imaging, graph theory, human connectome project, naturalistic stimulus

## Abstract

The extensive development of graph‐theoretic analysis for functional connectivity has revealed the multifaceted characteristics of brain networks. Network centralities identify the principal functional regions, individual differences, and hub structure in brain networks. Neuroimaging studies using movie‐watching have investigated brain function under naturalistic stimuli. Visual saliency is one of the promising measures for revealing cognition and emotions driven by naturalistic stimuli. This study investigated whether the visual saliency in movies was associated with network centrality. The study examined eigenvector centrality (EC), which is a measure of a region's influence in the brain network, and the participation coefficient (PC), which reflects the hub structure in the brain, was used for comparison. Static and time‐varying EC and PC were analyzed by a parcel‐based technique. While EC was correlated with brain activity in parcels in the visual and auditory areas during movie‐watching, it was only correlated with parcels in the visual areas in the retinotopy task. In addition, high PC was consistently observed in parcels in the putative hub both during the tasks and the resting‐state condition. Time‐varying EC in the parietal parcels and time‐varying PC in the primary sensory parcels significantly correlated with visual saliency in the movies. These results suggest that time‐varying centralities in brain networks are distinctively associated with perceptual processing and subsequent higher processing of visual saliency.

## Graphical abstract

While eigenvector centrality (EC) was correlated with brain activity in parcels in the visual and auditory areas during movie‐watching, it was only correlated with parcels in the visual areas in the retinotopy task. In addition, high participation coefficient (PC) was consistently observed in parcels in the putative hub both during the tasks and the resting‐state condition. Time‐varying EC in the parietal parcels and time‐varying PC in the primary sensory parcels significantly correlated with visual saliency in the movies. These results suggest that time‐varying centralities in brain networks are distinctively associated with perceptual processing and subsequent higher processing of visual saliency.

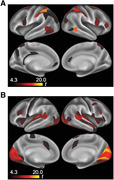

## 1 INTRODUCTION

In functional magnetic resonance imaging (fMRI) studies, movie‐watching is used as naturalistic stimuli rather than experimental stimuli. Movie‐watching evokes an “experimental” brain state as all participants receive similar stimuli, however, movie‐watching also induces a resting state as no response is required throughout the experiment. The brain activity during movie‐watching is usually analyzed with model‐based methods (e.g. motion, Bartels et al., 2008; binocular disparity, Ogawa et al., 2013), individual differences (Vanderwal et al., 2017), and inter‐subject correlation (Hasson et al., 2004, 2008; Betzel et al., 2020; Finn et al., 2020; for review, Pajula et al., 2012). Analyzing the brain activity associated with visual saliency is one of the potential measures for revealing cognitions and emotions driven by naturalistic stimuli (Bordier et al., 2013; Nguyen et al., 2017; for review, Sonkusare et al., 2019; Vanderwal et al., 2019).

The visual saliency of a scene characterizes spatially the strength of bottom‐up features. A visual saliency map computed with a biologically plausible model can encode the conspicuity of the visual scene and provide a prediction of the attention deployment (Itti et al., 1998; Itti & Koch, 2001; Harel et al., 2007). Visual saliency is known to modulate activation in higher‐order visual areas, and posterior parietal areas involved in visual attention (Gottlieb et al., 1998; Nardo et al., 2011, 2014; Capotosto et al., 2013; Santangelo & Macaluso, 2013; Santangelo et al., 2015). In addition to static scenes, visual saliency can serve to investigate the neural correlates of cognitive dynamics during movie‐watching (Nguyen et al., 2017).

The characteristics of large‐scale functional networks in the brain have been thoroughly investigated using graph‐theoretic analysis (Bullmore & Sporns, 2009; Rubinov & Sporns, 2010; Fornito et al., 2013, 2016; Bassett & Sporns, 2017). During movie‐watching, global and nodal graph indices characterize large‐scale functional networks (Kim et al., 2017). Among the several graph‐theoretic indices, network centrality identifies regions important for information processing in the brain network. While the eigenvector centrality (EC) indicates influential regions in the brain network both during tasks and in the resting state (Joyce et al., 2010; Lohmann et al., 2010; Zuo et al., 2012), the participation coefficient (PC) identifies the hub structure in the brain network (Sporns et al., 2007; Power et al., 2013; van den Heuvel & Sporns, 2013; Sporns, 2014). Therefore, high PC brain regions work as hubs for information processing, whereas high EC brain regions have a functional relevance for the brain network.

The large‐scale neural activity patterns underlying cognitive processes and behaviors are associated with time‐varying functional connectivity (Sakoğlu et al., 2010; Hutchison et al., 2013; Leonardi et al., 2014; Leonardi & Van De Ville, 2015; Lurie et al., 2020). Centrality changes along with temporary changes in functional connectivity depending on task demands. Recent investigations in time‐varying network centralities have revealed several brain network characteristics. Essentially, while the time‐varying EC of the resting‐state appears to reflect individual differences (Wink, 2019), time‐varying PC demonstrates the changes in the network structure with increasing integration of various brain regions (Thompson et al., 2020). These centralities represent the diverse characteristics of the brain network.

This study hypothesized that time‐varying centralities reveal brain network characteristics during movie‐watching and examined whether time‐varying EC and PC in the visual areas and posterior parietal regions correlated with visual saliency during movie‐watching. This study used fMRI data available from the public Human Connectome Project database (HCP, Uğurbil et al., 2013; Van Essen et al., 2013). Data pertaining to retinotopy task performance and the resting state were also analyzed for comparison. While the retinotopy task was expected to only evoke the activation of visual areas, no activation related to movie‐watching was expected during the resting‐state. HCP Parcellation (Glasser et al., 2016) was employed for centrality analysis. A parcel corresponded to a node in the brain network. The static (time‐averaged) and time‐varying EC and PC during movie‐watching, retinotopy task, and resting state were calculated parcel‐wise. Parcel‐based brain networks are preferred to voxel‐wise centrality analysis for analyzing time‐varying centrality since the latter involves a considerable amount of calculation load. Herein, the spatial map of PC was analyzed for consistency between movie‐watching, experimental task, and resting state. Time‐varying EC was used to evaluate the relationship between visual saliency and functional network profile during movie‐watching.

## 2 METHODS

### 2.1 Human connectome project data

Functional images were downloaded from the HCP database. The movie‐watching, retinotopy task, and resting‐state (both 7T and 3T images) data of 168 participants (64 males and 104 females; participants’ IDs are listed in Table S1) in various age groups (22–25 years: 19 participants; 26–30 years: 82; 31–35 years: 65; and ≥ 36 years: 2) were analyzed. Functional images were scanned using gradient‐echo echo‐planar imaging using a 7T scanner (Repetition Time (TR), 1000 ms; Echo Time (TE), 22.2 ms; Flip Angle, 45 degrees; Field of View, 208 mm × 208 mm; Voxel size, 1.6 mm isotropic; 85 slices; Multiband factor (Moeller et al., 2010), 5; Image acceleration factor, 2; Partial Fourier sampling, 7/8; Echo spacing, 0.64 ms; Band‐width, 1924 Hz/Px) and in a 3T scanner (TR, 720 ms; TE, 33.1 ms; Flip Angle, 52 degrees; Field of View, 208 mm × 180 mm; Voxel size, 2.0 mm isotropic; 72 slices; Multiband factor, 8; No image acceleration; Partial Fourier sampling, 7/8; Echo spacing, 0.58 ms; Band‐width, 2290 Hz/Px). The downloaded 7T data corresponded to the re‐processed fMRI data that were released in April 2018.

Movie‐watching (MOVIE hereafter), retinotopy task (RET hereafter), and resting‐state (REST hereafter) data were analyzed. The procedure for MOVIE and RET analysis included the following steps: For MOVIE, Creative Commons (CC), and Hollywood (HO) movies (Cutting et al., 2012) were presented on the screen (1024 × 768 resolution with 24 frames per second). Earbuds (Sensimetrics Corp., MA) were used to deliver the audio to participants. In the first fMRI run, CC movies were presented for a 15 min 21 s scan. In the second run, HO movies were presented for 15 min 18 s. In the third run, the different CC movies were presented for 15 min 15 s. In the fourth run, the different HO movies were presented for 15 min 01 s. There were rest phases (20 s) at the start and end of each fMRI run.

RET included six fMRI runs (Benson et al., 2018). Participants watched retinotopic stimuli videos, including counter‐clockwise sweep, clockwise sweep, expanding circle, contracting circle, and two multi‐direction bar sweeps. The stimuli were presented within a visual angle of 16° on the screen (1024 × 768 resolution with 15 frames per second). Each fMRI run lasted 5 min. Further details of MOVIE and RET tasks are described in the HCP reference manual chapter 2 (humanconnectome.org/study/hcp‐young‐adult/document/1200‐subjects‐data‐release).

### 2.2 Image processing

The image preprocessing details are described elsewhere (Glasser et al., 2013; Smith et al., 2013). Briefly, the following steps were performed: First, image susceptibility induced distortions were corrected. The images were spatially normalized into the standard space of Montreal Neurology Institute coordinates. Next, the data were normalized onto the standard surface (32,492 vertices in each hemisphere) (Van Essen, 2005; Glasser et al., 2013). For REST data, a temporal high‐pass filter (0.0005 Hz) was applied to remove the linear trend. FMRIB's ICA‐based Xnoiseifier (FIX, Salimi‐Khorshidi et al., 2014) was used to reduce noise and nuisance components automatically, such as, head motion. Multimodal surface matching was applied to adjust the normalization on the standard surface individually (MSMAll, Robinson et al., 2014, 2018). For MOVIE and RET data, a temporal high‐pass filter (0.005 Hz) was applied to remove low‐frequency fluctuations. The preprocessed files are available from the HCP web page (db.humanconnectome.org, RRID:SCR_004830).

**FIGURE 1:**
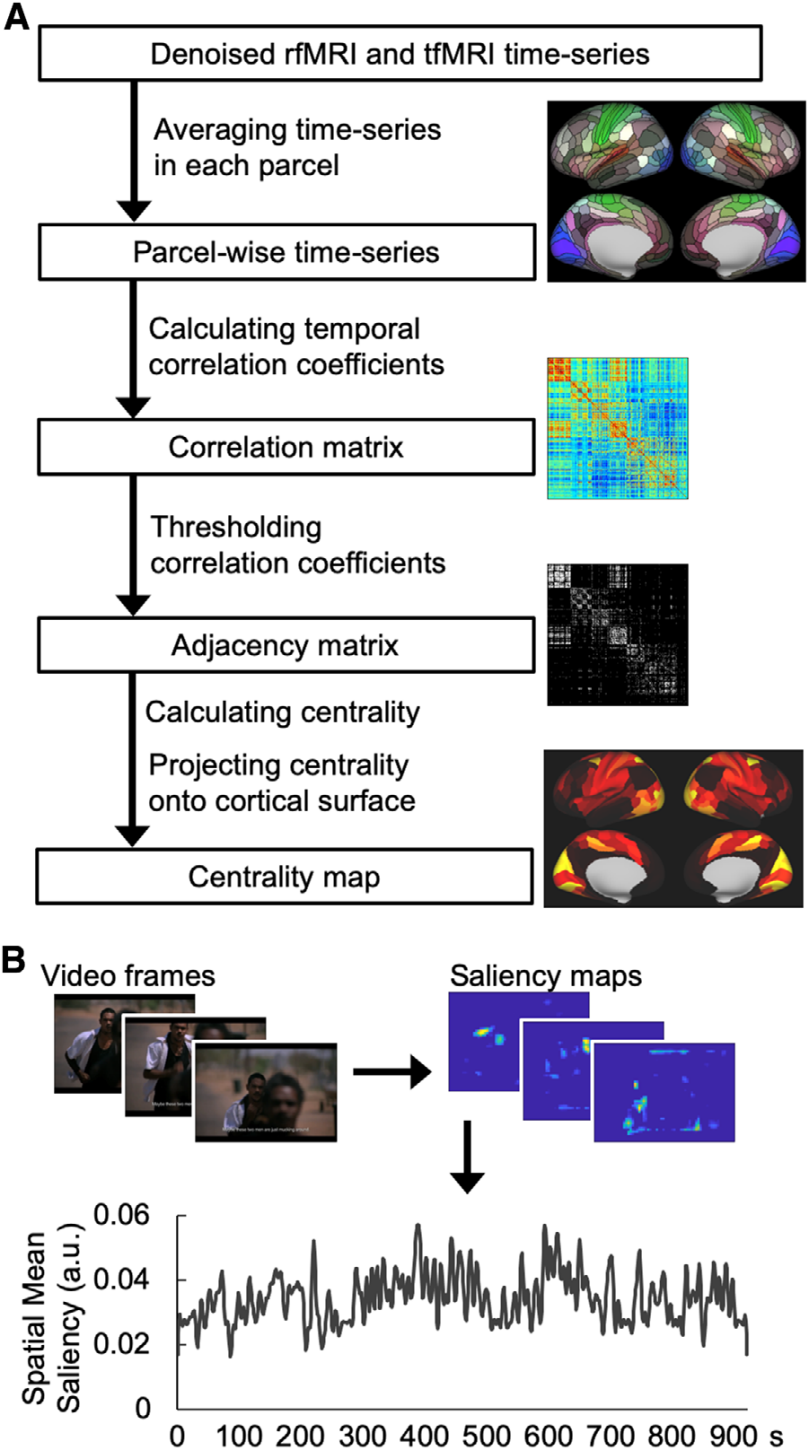
Procedure for calculating centrality map and visual saliency in movies. (A) Procedure for calculating centrality maps. The denoised functional magnetic resonance imaging (fMRI) time‐series are averaged in each parcel. Temporal correlation coefficients of the averaged time‐series are calculated between parcels. A threshold is applied to the correlation matrix to generate the adjacency matrix where each element indicates the presence or absence of connection between parcels. Then, network centrality in each parcel is calculated and projected onto the standard brain surface to generate a centrality map. (B) Calculation of visual saliency in movies. The visual saliency map of each movie frame is calculated. The maps within each scan are spatially averaged to generate the mean visual saliency signal. A low‐pass filter is applied to the mean visual saliency signal

Figure 1A shows the procedure to calculate a centrality map on the standard brain surface. The time‐series of the preprocessed data were averaged across vertices in each parcel. For this purpose, the HCP parcellation, including 180 parcels in each hemisphere (Glasser et al., 2016), was employed. The averaged time‐series in each parcel for each fMRI run was normalized to mean and standard deviation values of 0 and 1, respectively. The time‐series were subsequently concatenated across the fMRI runs of each participant for centrality analysis. A correlation matrix was calculated for the concatenated time‐series across all parcels.

Midnight Scan Club scripts (github.com/MidnightScanClub, Gordon et al., 2017) were used to read and write CIFTI files. The HCP Connectome Workbench (Marcus et al., 2011, RRID:SCR_008750) was used to calculate the correlation matrix and visualize the results.

### 2.3 Network analyses

A proportional 7.5% threshold was applied to the correlation matrix to generate an adjacency matrix, which represented a binary undirected network (van den Heuvel et al., 2017; Figure 1). The adjacency matrix was used for calculating EC and PC. The threshold was determined based on the flow coefficient (Honey et al., 2007). As opposed to EC and PC, the flow coefficient is a local centrality metric. With respect to the network construction, the network nodes are expected to influence or communicate with each other more intensively. The mean flow coefficients across participants were calculated at 2.5%, 5.0%, 7.5%, 10.0%, 12.5%, and 15% proportions. The peak was found at 7.5%.

EC evaluates the importance of each network node—parcel—(Lohmann et al., 2010; Zuo et al., 2012). All connections in the adjacency matrix were used to calculate EC. Therefore, EC integrates the connectivity information about all functionally connected parcels. EC can be used to capture brain‐wide large‐scale characteristics. Thus, a high EC brain region is functionally crucial in the brain network (e.g. network resilience). The EC of parcel *i* was calculated as below:

(1) $$\;EC_{i}=\frac{1}{\lambda_{1}}\;A\;\mu_{1}=\frac{1}{\lambda_{1}}\;\sum_{j\;=\;1}^{N}a_{ij}\mu_{1}\left(j\right)$$

***A*** is the adjacency matrix, *λ*_1_ is the first eigenvalue, ***μ*_1_** is the first eigenvector, and *N* is the number of parcels. PC of parcel *i* was also calculated for the brain network (Guimerà & Nunes Amaral, 2005; Power et al., 2013):

(2) $$\;PC_{i}=\;1-\sum_{m\in M}{\left(\frac{K_{i}\left(m\right)}{K_{i}}\right)}^{2}$$

*M* is the total set of network communities estimated using Louvain community detection (Rubinov & Sporns, 2010), *K_i_* is the number of connections associated with parcel *i*, and *K_i_* (*m*) is the number of connections between parcel *i* and all parcels in community *m*. PC can be used to define a parcel acting as a connector hub between modules (i.e. local sub‐networks) (Guimerà & Nunes Amaral, 2005; Rubinov & Sporns, 2010; Bertolero et al., 2015; Cohen & D'Esposito, 2016). While parcels with higher PC have connections across modules, parcels with lower PC tend to have connections within a module. In contrast to that of EC, the spatial profile of PC, that reflects the hub structure in the brain, was consistent between the tasks and resting‐state condition.

Both time‐varying eigenvector centrality (tEC) and participation coefficient (tPC) were calculated using the sliding window technique (Shakil et al., 2016). The window was set to 50 s (from before 25 s to after 25 s). The tEC and the tPC were calculated every second in time with the scan TR in MOVIE, except the first and the last 25 s. At time *t*, the adjacency matrix ***A***(*t*) was calculated. Then, the tEC of parcel *i* at time *t* was calculated as below:

(3) $$\mathrm{tE}C_{i}\;\left(t\right)=\frac{1}{\lambda_{1}\left(t\right)}\;A\left(t\right)\;\mu_{1}\;\left(t\right)=\frac{1}{\lambda_{1}\left(t\right)}\;\sum_{j\;=\;1}^{N}a_{ij}\left(t\right)\;\mu_{1}\left(j,\;t\right)$$

The first eigenvalue *λ*_1_(*t*) and first eigenvector ***μ*_1_**(*t*) were calculated from the adjacency matrix ***A***(*t*). The tPC of parcel *i* was also calculated as below:

(4) $$\mathrm{tP}C_{i}\;\left(t\right)=\;1-\sum_{m\in M\left(t\right)}{\left(\frac{K_{i}\left(m,t\right)}{K_{i}\left(t\right)}\right)}^{2}$$

The total set of network communities of time *t*, *M*(*t*), was estimated from the adjacency matrix ***A***(*t*).

The brain connectivity toolbox (Rubinov & Sporns, 2010, RRID:SCR_004841) was used to calculate flow coefficient, EC, PC, tEC, and tPC.

### 2.4 Visual saliency

The saliency toolbox (version 2.3, saliencytoolbox.net; Walther & Koch, 2006) was used to calculate the visual saliency map (Itti et al., 1998) of every movie frame (Figure 1B). Visual saliency was calculated as follows. Each movie frame was decomposed into image features: intensity, color, and orientation. Feature maps were computed from the Gaussian pyramid procedure using the center‐surround mechanism. Then, each feature map was normalized. Finally, the visual saliency map was obtained by combining the feature maps. The visual saliency map of each movie frame was 64 pixels in width and 45 pixels in height.

The visual saliency maps within each fMRI scan were spatially averaged across pixels and temporally averaged across frames to generate the mean visual saliency signal over time for each scan, as follows:

(5) $$\bar{s}\;\left(t\right)=\frac{1}{F}\;\frac{1}{X}\frac{1}{Y}\sum_{f\in t}\sum_{x}\sum_{y}s\left(x,y,f\right)$$

*F* indicates the number of frames in time *t* (i.e. 24 frames), *X* indicates the number of pixels in the x‐axis in the visual saliency map, and *Y* indicates the number of pixels in the y‐axis in the visual saliency map. A low‐pass filter (0.1 Hz) was applied to the mean visual saliency signal. FMRI signals in visual areas were expected to reflect visual saliency. The correlation between the fMRI signal in each parcel and the mean visual saliency signal was examined.

### 2.5 Parcel‐wise relations between visual saliency and time‐varying centralities

The temporal correlation between the mean visual saliency signal and tEC was calculated for each parcel in each participant. The temporal correlation between the mean visual saliency signal and tPC was also calculated. Subsequently, the correlation coefficients were transformed to Fisher‐z value and statistically compared for each parcel. Bonferroni correction was applied for the number of parcels, although the Bonferroni correction might be conservative and cause false negatives. Statistical threshold was set to Z > 4.3 (*P*‐FWE < 0.01).

## 3 RESULTS

### 3.1 Network construction

**FIGURE 2:**
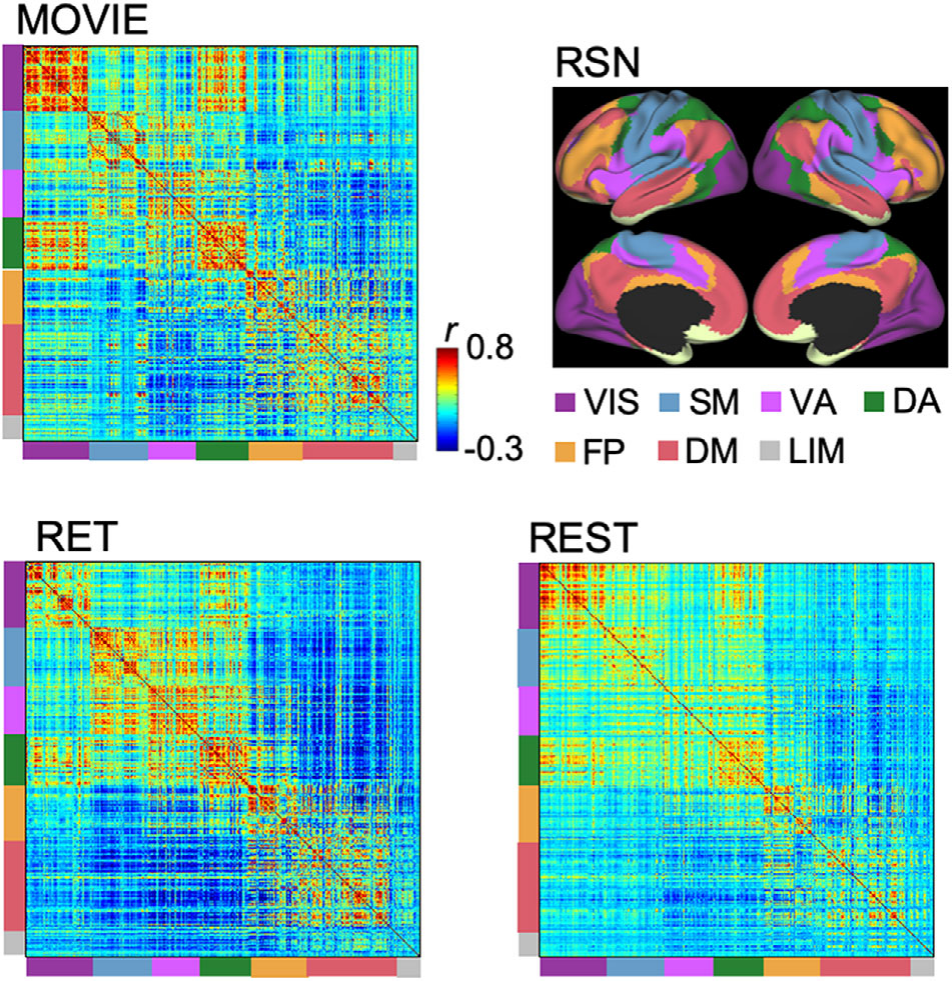
Correlation matrices in MOVIE, RET, and REST aligned with Yeo's seven RSNs. A threshold is applied to these matrices to generate the networks for calculating centralities. rfMRI, resting‐state functional magnetic resonance imaging; tfMRI, task‐based fMRI; VIS, visual; SM, somatomotor; VA, ventral attention; DA, dorsal attention; FP, fronto‐parietal; DM, default mode; LIM, limbic; RSNs, resting‐state networks; MOVIE, movie‐watching; RET, retinotopy task, REST, resting‐state

Figure 2 shows the correlation matrices of MOVIE, RET, and REST. The parcels were aligned for Yeo's seven resting‐state networks (RSN, Yeo et al., 2011). Applying the proportional threshold to the correlation matrices generated adjacency matrices showing dense connections in each RSN, and sparse connections between RSNs (Figure S1). Each adjacency matrix represents the brain network for each of MOVIE, RET, and REST.

### 3.2 Centralities

**FIGURE 3:**
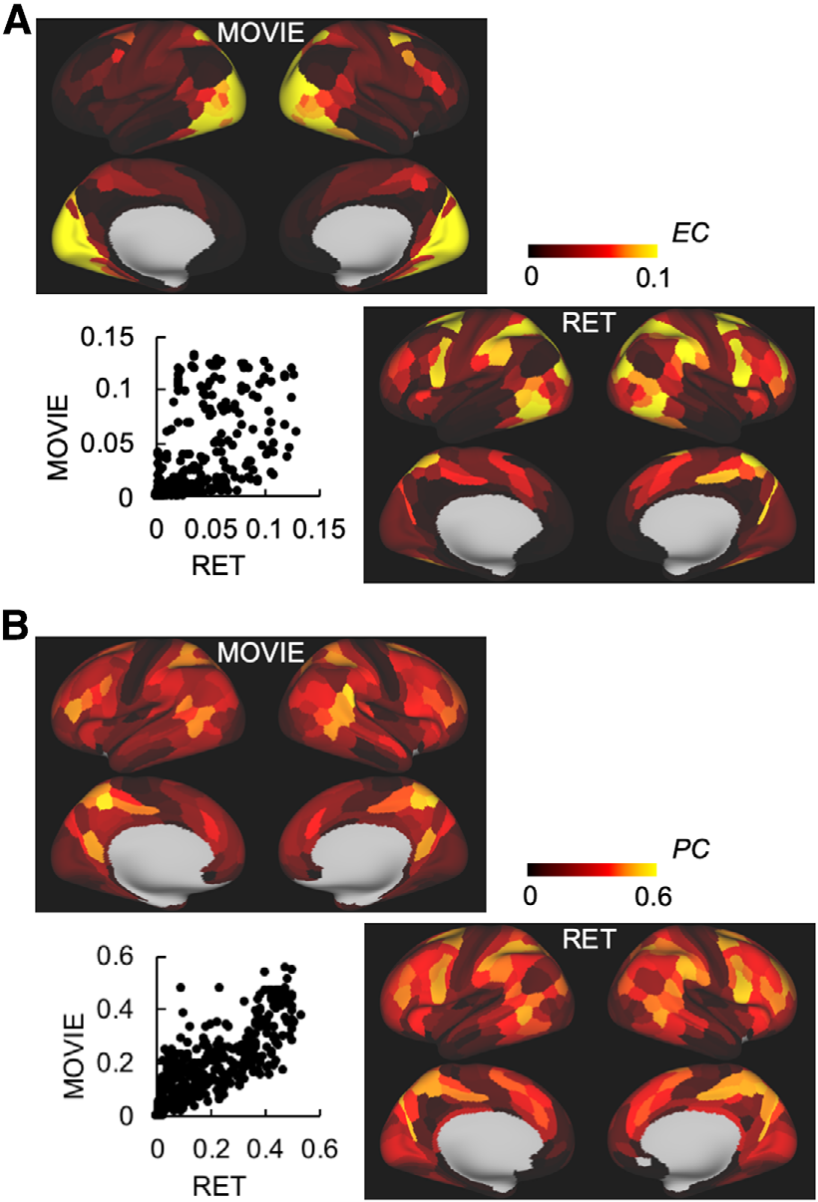
Relationship of centralities between MOVIE, RET, and REST. (A) EC maps. EC maps in MOVIE and RET are spatially correlated. Each dot represents a parcel. (B) PC maps. PC maps in MOVIE and RET are spatially correlated. EC, eigenvector centrality; PC, participation coefficient; MOVIE, movie‐watching; RET, retinotopy task, REST, resting‐state

EC and PC were calculated for each parcel, and for MOVIE, RET, and REST. Figure 3A shows the EC maps for MOVIE and RET. The correlation coefficient between MOVIE and RET was 0.60. Figure 3B shows the PC maps for MOVIE and RET. The correlation coefficient between MOVIE and RET was 0.78. The correlation for PC was significantly higher than the correlation for EC (Z = 3.28, *P* = 0.001). EC and PC maps for REST are shown in Figure S2. Correlations of MOVIE‐REST and RET‐REST with MOVIE‐RET were consistently higher (0.73 and 0.85) for PC, and also for EC (0.80 and 0.64) (Figure S2). These indicate that the spatial distribution of PC was consistent during movie‐watching, experimental task, and resting state.

**FIGURE 4:**
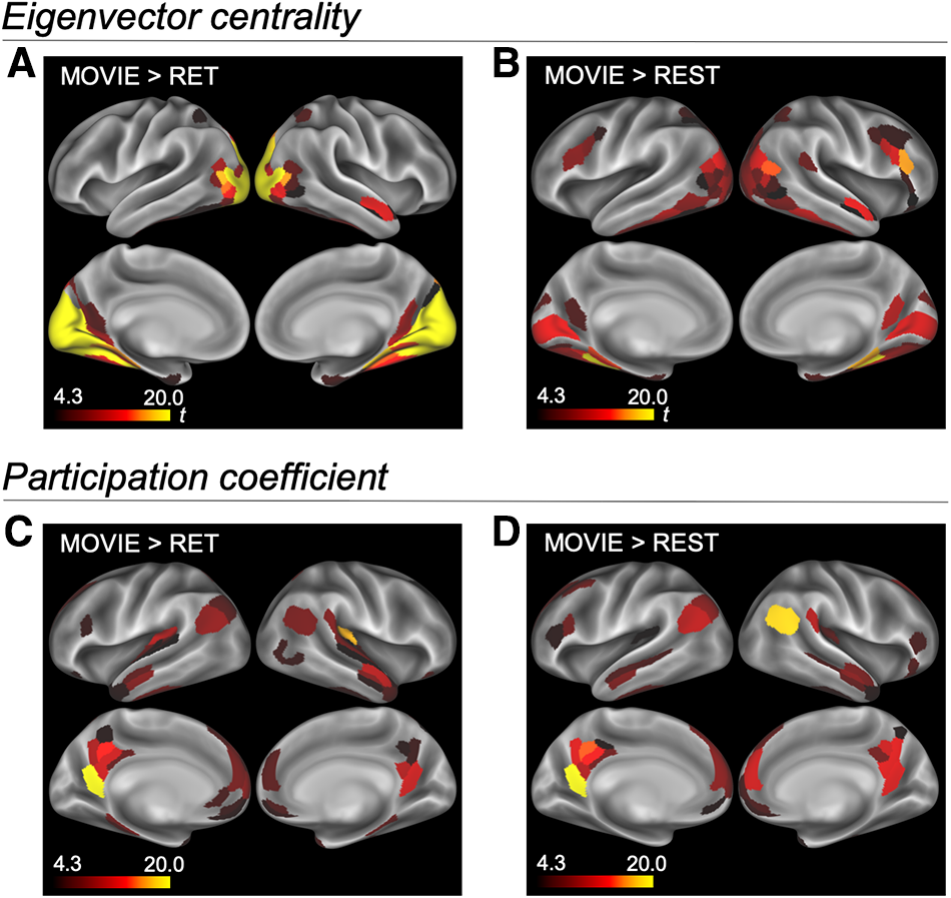
Comparisons of EC and PC in each parcel. (A) Result of MOVIE > RET of EC. Parcels in visual areas show significantly higher EC for MOVIE. (B) Result of MOVIE > REST of EC. Parcels in visual areas also show significantly higher EC for MOVIE. (C) Result of MOVIE > RET of PC. Parcels in the default‐mode network show significantly higher EC for MOVIE. (D) Result of MOVIE > REST of PC. Parcels in the default‐mode network also show significantly higher EC for MOVIE. EC, eigenvector centrality; PC, participation coefficient; MOVIE, movie‐watching; RET, retinotopy task, REST, resting‐state

ECs in MOVIE were compared with those in RET and REST for each parcel. The parcels in visual areas showed higher EC for MOVIE compared with RET and REST (Figure 4A,B, Table S2; paired t tests with Bonferroni correction for the number of parcels). The parcels in the inferior parietal lobule, lateral prefrontal cortex, and medial prefrontal cortex had higher EC for RET compared with MOVIE and REST (Figure S3A,B, Table S6). The parcels in the somatomotor network showed higher EC for REST compared with MOVIE and RET (Figure S3C,D).

Likewise, PCs in MOVIE were compared with those in RET and REST for each parcel. Although the PC maps were similar among MOVIE, RET, and REST, the parcels in the default‐mode network showed significantly higher PC for MOVIE compared with RET and REST (Figure 4C,D, Table S3; paired t tests with Bonferroni correction for the number of parcels). The other comparisons are shown in Figure S4 (see also Table S7).

We also examined whether the head motion influenced network centrality. The average head motion in each fMRI run was available from the HCP database (file: Movement_RelativeRMS_mean.txt). The mean head motion was calculated across fMRI runs for each participant in each condition. The correlation between mean head motion and network centralities across participants was calculated in each parcel. The results showed low correlations between head motion and network centralities (Figure S5).

### 3.3 Visual saliency

**FIGURE 5:**
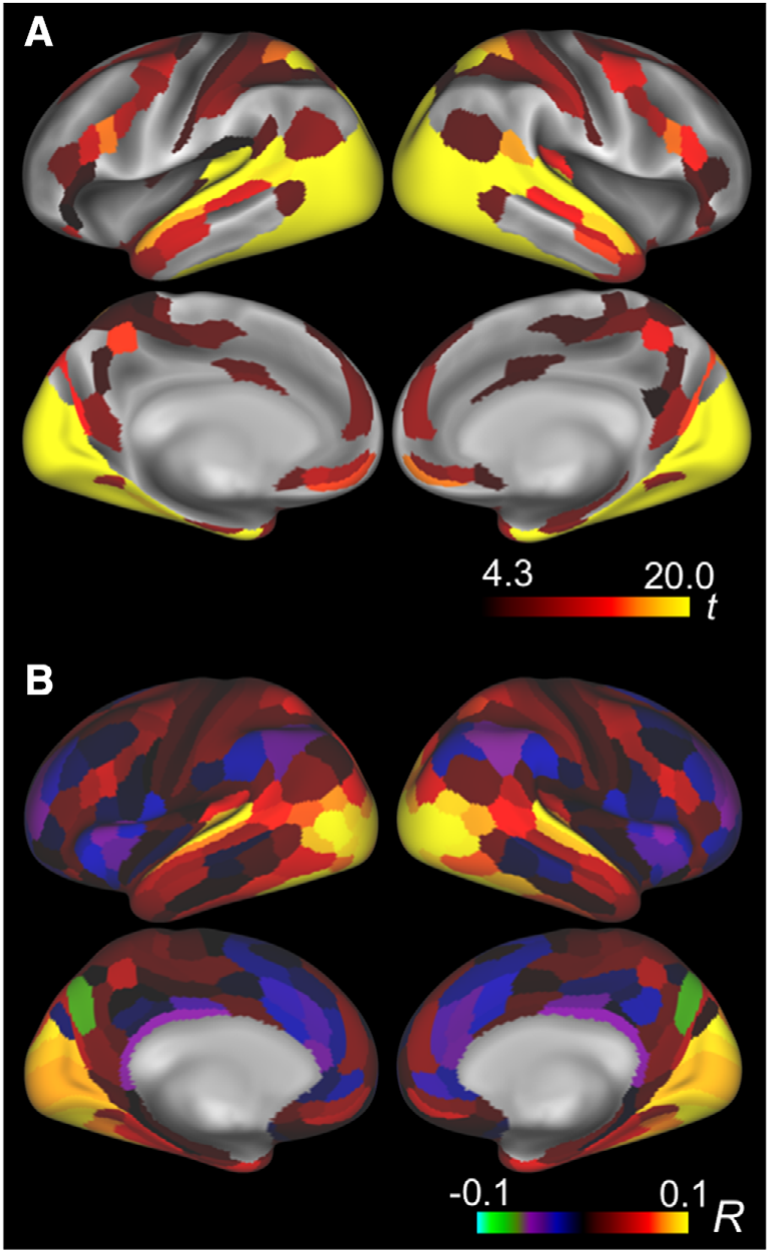
Parcels associated with visual saliency. (A) Parcels correlated with the mean visual saliency signal. Parcels in the visual and auditory areas show significant correlations. (B) Group‐averaged r map. The Fisher‐z‐transformed correlation in each parcel was averaged across participants and Fisher‐z‐inverse‐transformed into the group‐average correlation coefficient

The correlation coefficients between the fMRI signal in each parcel and the mean visual saliency signal were Fisher‐z transformed and statistically tested (one sample t test). As expected, the fMRI signals in the parcels in visual areas were significantly correlated with the mean visual saliency signal (Figure 5A, Table S4; paired t‐test with Bonferroni correction for the number of parcels). Significant correlations were also observed in parcels in the auditory areas. The visual saliency could emphasize the auditory signals. Figure 5B shows the group‐averaged r‐map of the same correlation. The averaged correlation coefficient was higher in parcels in the visual areas.

### 3.4 Relationship between visual saliency and time‐varying eigenvector centrality and participation coefficient

**FIGURE 6:**
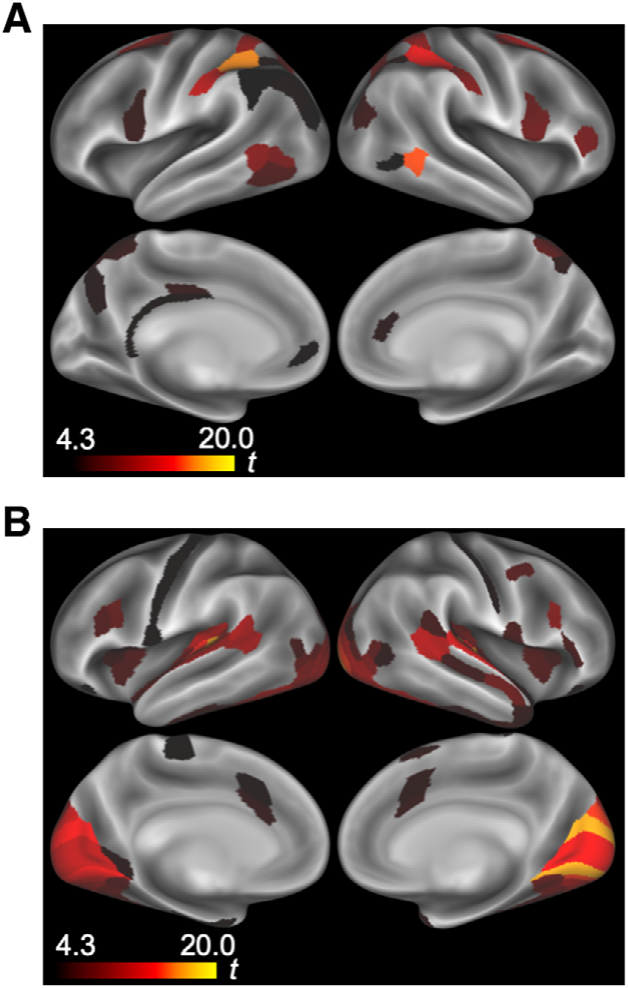
Parcels with higher correlations between visual saliency and time‐varying network centrality. (A) Contrast of Fisher‐z‐transformed correlations of tEC > tPC with visual saliency. Posterior parietal parcels show a higher correlation between tEC and visual saliency. (B) Contrast of Fisher‐z‐transformed correlation of tPC > tEC with visual saliency. Parcels in primary sensory areas show a higher correlation between tPC and visual saliency. tEC, time‐varying eigenvector centrality; tPC, time‐varying participation coefficient

The mean saliency signal was calculated for each TR scan and temporally filtered (Low‐pass 0.1 Hz). Correlations between mean visual saliency signal, and tEC and tPC, were calculated for each parcel. Fisher‐z‐transformed correlations were compared in each parcel (paired t test with Bonferroni correction for the number of parcels). The posterior parietal parcels showed a significant correlation between saliency and tEC (Figure 6A, Table S5), while the primary sensory area parcels showed a significant correlation between saliency and tPC (Figure 6B, Table S5).

## 4 DISCUSSION

This study investigated the association between time‐varying network centrality in the brain and visual saliency during movie‐watching. A parcel‐based technique was used to construct a brain network from the functional connectivity between parcels. When comparing MOVIE and RET, the PC maps were similar, while the spatial distributions of EC were less similar. The correlation analysis between visual saliency and time‐varying network centralities showed that tEC in the posterior parietal parcels, and tPC in the primary sensory areas, tracked visual saliency. These results suggest that tEC and tPC may be associated with different perceptual features of visual saliency.

The parcels in the posterior parietal cortex showed a significant correlation between tEC and visual saliency. Previous studies have demonstrated the role of the posterior parietal cortex in higher processing for visual saliency (Gottlieb et al., 1998; Arcizet et al., 2011; Santangelo et al., 2015; Chen et al., 2020). The results of this study suggest that large‐scale brain networks are associated with visual saliency processing: The ventral attention network for stimulus‐driven attentional capture (Yantis & Egeth, 1999) and the dorsal attention network for top‐down attentional direction to contextually relevant objects (Connor et al., 2004). Consequently, time‐varying network centralities can be used to characterize the profile of large‐scale brain activity. However, large motion in movie frames may influence attention and eye movement, a fact to be considered when viewing this sort of data.

While in MOVIE EC was high in visual areas, in RET it was high in parcels in the ventral attention and somatomotor networks (Figure 4). These may reflect the task features of MOVIE and RET conditions. Compared with MOVIE, RET required pressing a button when the cue appeared. To detect the cue quickly, the activity in the parcels in the ventral attention network may correlate with the cue appearance. The parcels in the somatomotor network may increase the activity in a synchronized manner to prepare for the button press.

The spatial distribution of PC was highly consistent in MOVIE, RET, and REST. The correlation for PC was significantly higher than that for EC in MOVIE. In addition, although the magnitude of the static magnetic field and other variations in scan settings may have influenced functional connectivity, PC was found to be consistent in the resting state in the different static magnetic fields of 3T and 7T (Figure S6). These results indicate that PC reflects the stable hub structure and may be related to anatomical connections (Power et al., 2013; van den Heuvel & Sporns, 2013). A set of connected parcels with high PC called rich‐club nodes may play an important role in information integration and network robustness (van den Heuvel & Sporns, 2011; Crossley et al., 2013). Complementary to PC, which can detect a parcel acting as a connector hub between modules, within‐module degree z‐score can be used to detect a parcel with connections within a module (i.e. provincial hub) (Guimerà & Nunes Amaral, 2005; Rubinov & Sporns, 2010; Baum et al., 2017). To reveal the functional integration and segregation of brain networks, the combination of complemental network indices can be considered.

This study adopted a parcel‐based technique to analyze the network centrality during movie‐watching. Parcel‐based analysis is becoming popular, not only for the resting state but for task‐induced activation (Allan et al., 2019; Osada et al., 2019; Fujimoto et al., 2020; Suda et al., 2020). The main benefit of the parcel‐based analysis, over voxel‐wise or vertex‐wise analysis of the whole brain, is lower computational load in both time and memory. However, one important limitation of the parcel‐based analysis is its lower spatial resolution. Nevertheless, as a tool for analysis of the spatial distribution of network centrality in the whole brain, as in this case, the parcel‐based analysis is more suitable than the vertex‐wise analysis.

Time‐varying network centrality may be beneficial for characterizing brain activity (Calhoun et al., 2014). The dynamics of network centricity calculated from functional connectivity are greatly affected by the nature of time‐varying functional connectivity. In this study, the correlation between tEC and visual saliency was significant mainly in the posterior parietal cortex. In contrast, a higher EC was observed in the early visual cortex. The correlation between tPC and visual saliency was significant in primary sensory areas, whereas a higher PC was observed mainly in the prefrontal and posterior parietal cortices. Figure 6 shows significant parcels where centrality changes as visual saliency changes. tPC being higher in the visual cortex when visual saliency is also high likely reflects the fact that information is sent from the visual cortex to various areas when visual saliency is increased. Thus, time‐varying network centrality may not be related to the nature of static (or time‐averaged) network centrality. Caution should be exercised in the interpretation of time‐varying centrality, as its polarities are temporally not always equal to those of static network centrality (Thompson et al., 2020).

Many resting state functional connectivity studies of brain networks use the Pearson's product‐moment correlation coefficient and Fisher‐z‐transformation. When time series of the fMRI signals exhibit high temporal autocorrelation, the effective degrees of freedom are reduced and the standard error may be biased. Therefore, care should be taken when assessing the effective degrees of freedom influencing the network formation. The proportional threshold would be less sensitive to the effective degrees of freedom than the absolute threshold. A solution would thus be to use the effective degrees of freedom for the Pearson's correlation that considers the temporal autocorrelation of the time series (Afyouni et al., 2019).

This study investigated the relationship between time‐varying network centrality and visual saliency during movie‐watching. The results showed a significant correlation between visual saliency and tEC in posterior parietal parcels and between visual saliency and tPC in primary sensory area parcels. The results also suggest that tEC is associated with spatial attention control in the parietal cortex evoked by visual saliency. These findings suggest that network centralities can be used for investigating brain activity characteristics in response to time‐varying stimulus features, albeit with some caution in the interpretation.

## COMPETING INTERESTS

The author declares no competing interests.

### PEER REVIEW

The peer review history for this article is available at https://publons.com/publon/10.1002/brb3.2334.

## Supporting information

SUPPORTING INFORMATION

## Data Availability

The centrality maps and the statistical results on the standard surface will be made available on reasonable request.
