# Supplementary material for: Time‐varying measures of cerebral network centrality correlate with visual saliency during movie watching
Source: Brain Behav. 2021 Aug 26;11(9):e2334. doi: 10.1002/brb3.2334 (PMC8442596; doi:10.1002/brb3.2334)
Supplement: Supplementary file 1 — SUPPORTING INFORMATION [file BRB3-11-e2334-s001.docx]

**Supplementary Information**

**Time-varying measures of cerebral network centrality correlate with visual saliency**

**during movie watching**

**Akitoshi Ogawa**

Faculty of Medicine, Juntendo University

2-1-1 Hongo, Bunkyo-ku, Tokyo 113-8421, Japan

a-ogawa@juntendo.ac.jp

**
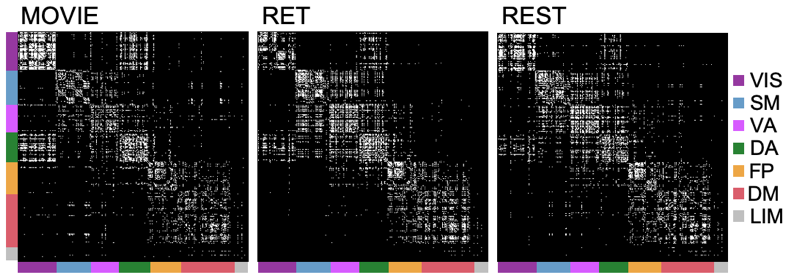
**

**Figure S1.** Adjacency matrices aligned with Yeo’s seven resting-state networks in MOVIE, RET, and REST experiments. All adjacency matrices show dense connections within each RSN but sparse connections among resting-state networks. VIS, visual; SM, somatomotor; VA, ventral attention; DA, dorsal attention; FP, fronto-parietal; DM, default mode; LIM, limbic; RSNs, resting-state networks; MOVIE, movie-watching; RET, retinotopy task, REST, resting-state.


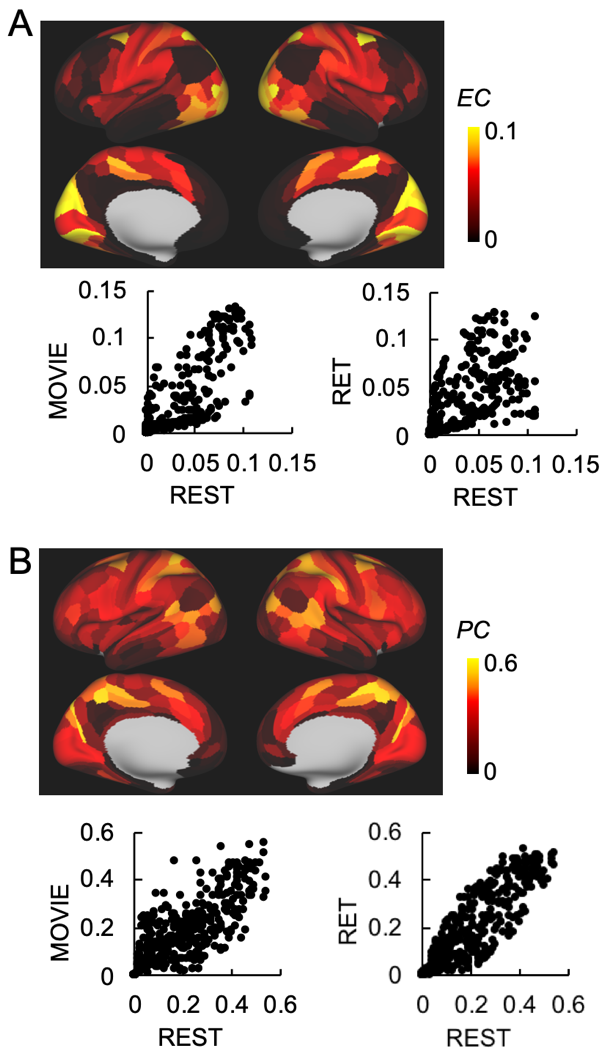


**Figure S2.** Relationship between MOVIE and REST, and between RET and REST in EC and PC. **A.** EC map in REST. The EC in REST correlates with that in MOVIE and in RET. **B.** PC map in REST. The PC in REST correlates highly with that in MOVIE and in RET. MOVIE, movie-watching; RET, retinotopy task, REST, resting-state; EC, eigenvector centrality; PC, participation coefficient.

**
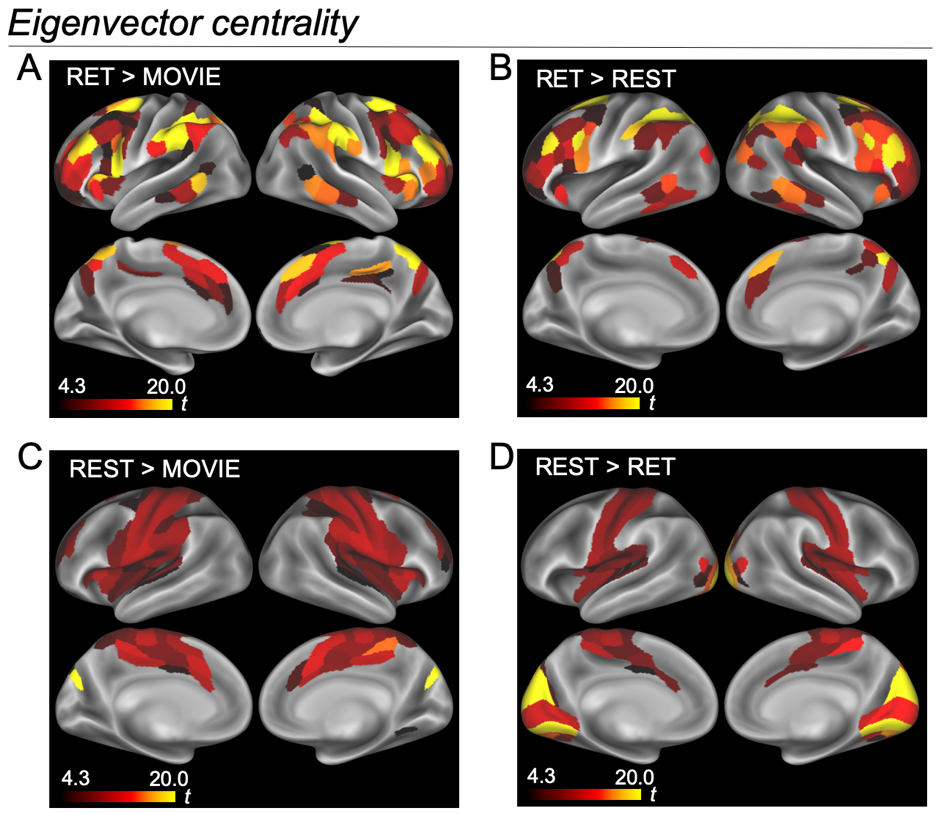
**

**Figure S3.** EC comparisons between RET and others, and between REST and others (paired ttests with Bonferroni correction for the number of parcels). The panels show parcels with significantly higher EC in RET than in MOVIE (**A**), in RET than in REST (**B**), in REST than in MOVIE (**C**), and in REST than in RET (**D**). EC, eigenvector centrality; MOVIE, movie-watching; RET, retinotopy task, REST, resting-state. See Table S6 for statistics.


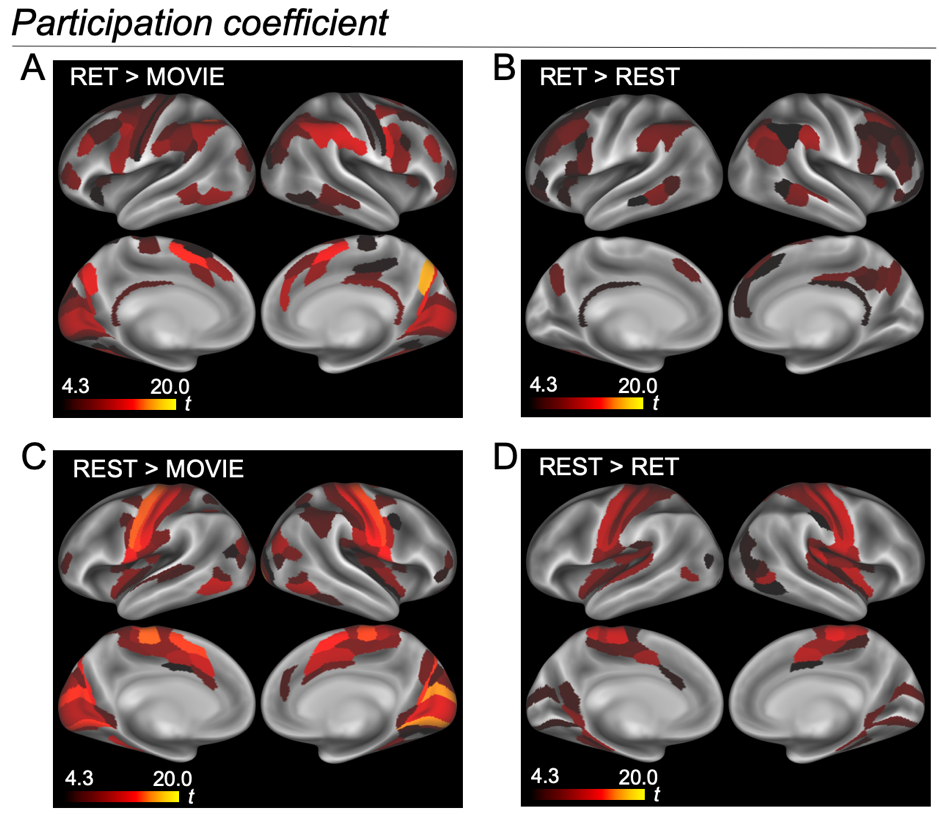


**Figure. S4.** PC comparisons between RET and others, and between REST and others (paired t tests with Bonferroni correction for the number of parcels). The panels show parcels with significantly higher PC in RET than in MOVIE (**A**), in RET than in REST (**B**), in REST than in MOVIE (**C**), and in REST than in RET (**D**). PC, participation coefficient; MOVIE, movie-watching; RET, retinotopy task, REST, resting-state. See Table S7 for statistics.


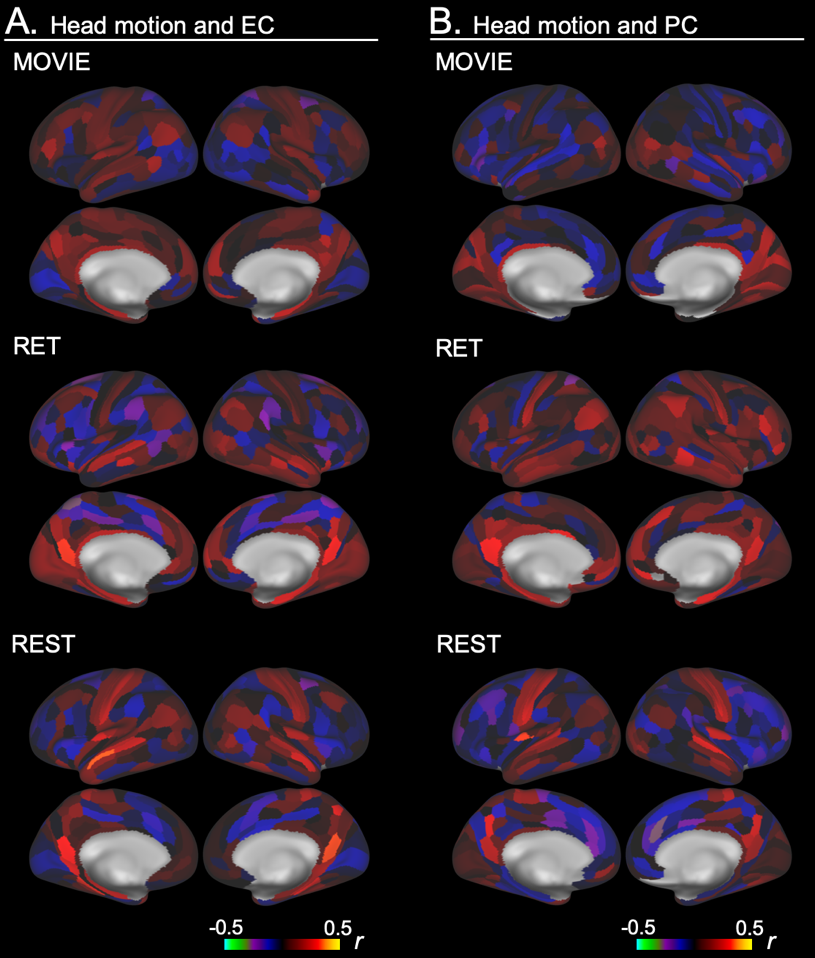


**Figure S5.** Relations between head motion and the EC and PC in MOVIE, RET, and REST. **A.** Correlation maps between head motion and EC. **B.** Correlation maps between head motion and PC. The group averages of head motions were respectively 0.110 ± 0.046, 0.105 ± 0.047, 0.063 ± 0.027 (mean ± standard deviation) in MOVIE, RET, and REST. The head motions in MOVIE and RET were significantly more prominent than in REST. The head motion in MOVIE was slightly larger than that in RET. MOVIE, movie-watching; RET, retinotopy task, REST, resting-state; EC, eigenvector centrality; PC, participation coefficient.


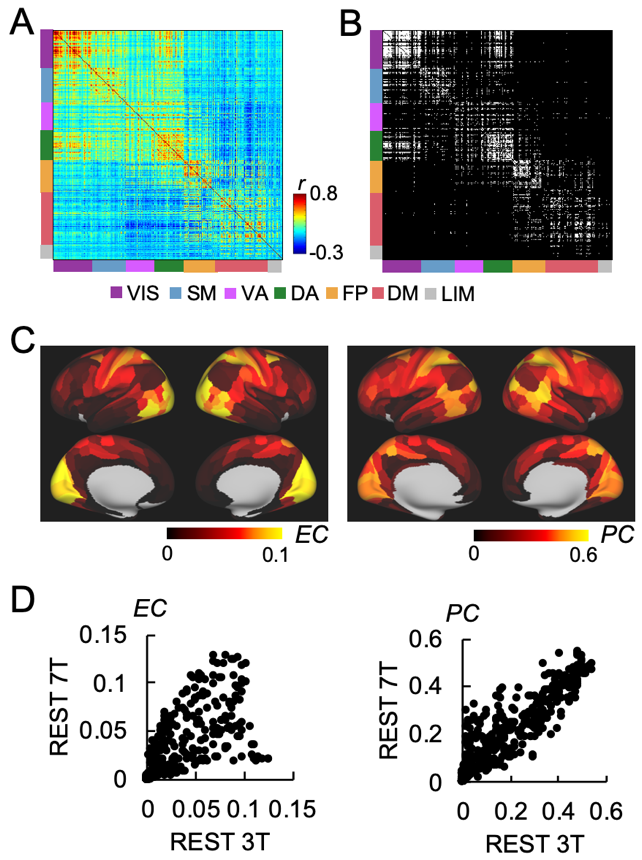


**Figure S6.** Consistency of EC and PC between REST in 3T and 7T. **A.** Correlation matrix of REST in 3T aligned with the seven resting-state networks. **B.** Adjacency matrix calculated from the correlation matrix. **C.** EC map (left) and PC map (right) in REST in 3T. **D.** Spatial correlations of EC and PC between REST in 3T and 7T (EC, *r* = 0.69; PC, *r* = 0.875). Each dot indicates a parcel. These show the high consistency of PC for REST in 3T and 7T. In addition, EC is also consistent between 3T and 7T. These indicate that the results of the centralities in 7T can be extended to 3T data. VIS, visual; SM, somatomotor; VA, ventral attention; DA, dorsal attention; FP, fronto-parietal; DM, default mode; LIM, limbic; MOVIE, movie-watching; RET, retinotopy task, REST, resting-state; EC, eigenvector centrality; PC, participation coefficient.


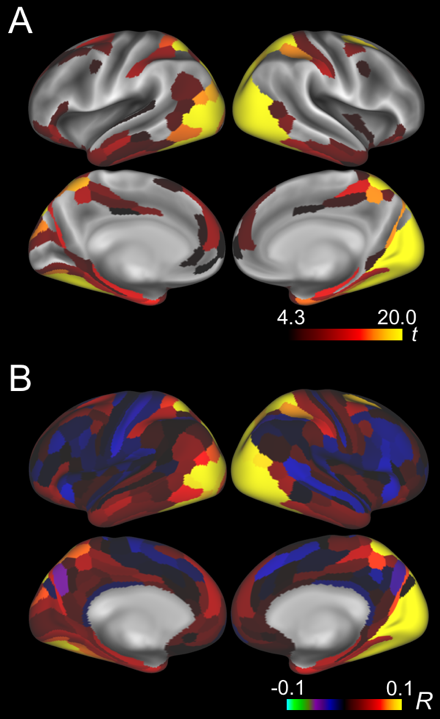


**Figure S7.** Parcels associated with visual saliency in RET. **A.** Parcels correlated with the mean visual saliency signal in RET. Parcels in visual areas primarily show significant correlations with the mean visual saliency. **B.** Group-averaged r-map in RET. The Fisher-z-transformed correlation in each parcel was averaged across participants and Fisher-z-inverse-transformed into the group-average correlation coefficient.


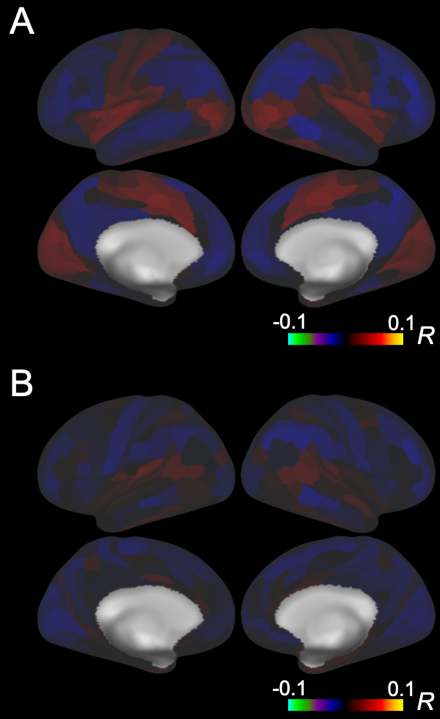


**Figure S8.** Relationship between time-varying centrality and head motion (HM). **A.** Relation between tEC and HM in each parcel (r map). The correlation coefficients were Fisher-z-transformed and averaged across participants in each parcel. The average was then Fisher-z-inverse-transformed into the group-average correlation coefficient. The maximum is 0.023. **B.** Relation between tPC and HM in each parcel (r map). The group-average correlation coefficient was calculated as tEC. The maximum is 0.014.


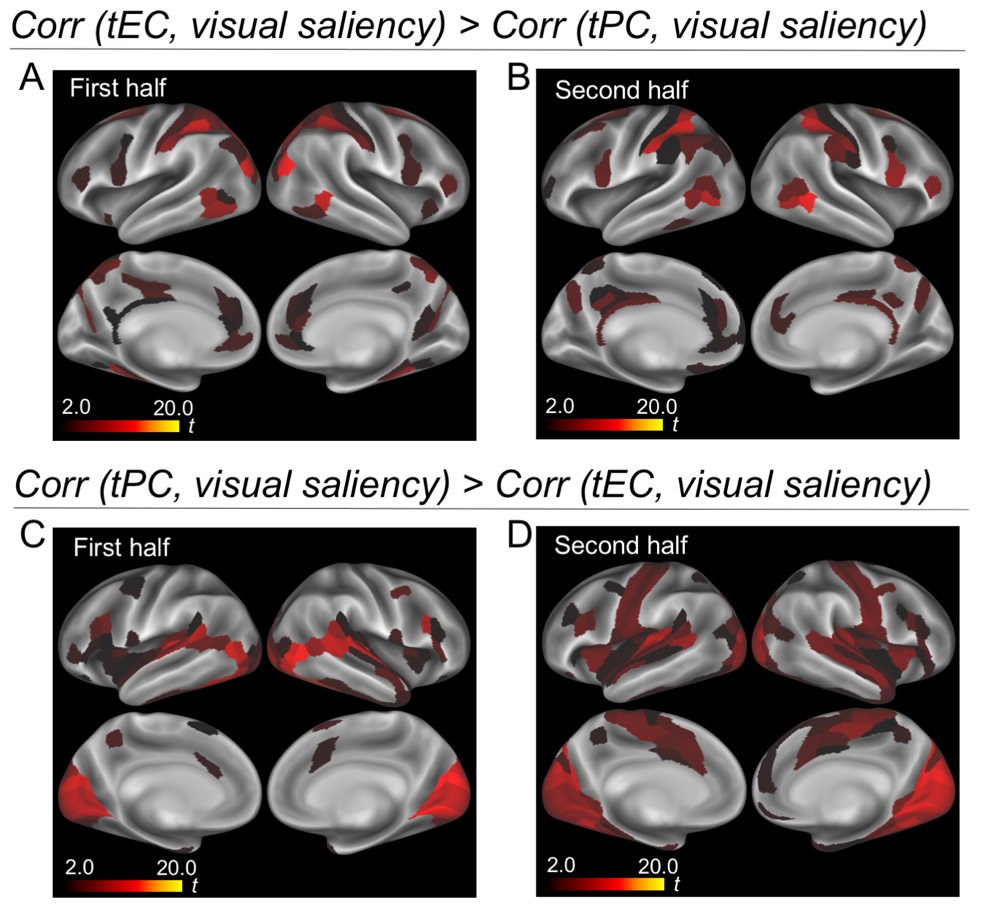


**Figure S9.** Reproducibility of correlations between the time-varying centrality and visual saliency. **AB.** Reproducibility of correlation between visual saliency and eigenvector centrality.

**Table S1.** List of participants’ IDs.

| 100610 | 102816 | 104416 | 105923 | 108323 | 109123 | 114823 | 115017 | 115825 | 116726 |
| --- | --- | --- | --- | --- | --- | --- | --- | --- | --- |
| 118225 | 125525 | 126426 | 128935 | 130114 | 130518 | 131217 | 131722 | 132118 | 134627 |
| 134829 | 135124 | 137128 | 140117 | 144226 | 145834 | 146129 | 146432 | 146735 | 146937 |
| 148133 | 150423 | 155938 | 156334 | 157336 | 158035 | 158136 | 159239 | 162935 | 164131 |
| 164636 | 165436 | 167036 | 167440 | 169040 | 169343 | 169444 | 169747 | 171633 | 172130 |
| 173334 | 175237 | 176542 | 177140 | 177645 | 177746 | 178142 | 178243 | 178647 | 180533 |
| 181232 | 182436 | 182739 | 185442 | 187345 | 191033 | 191336 | 191841 | 192439 | 192641 |
| 193845 | 195041 | 196144 | 197348 | 198653 | 199655 | 200311 | 200614 | 201515 | 203418 |
| 204521 | 205220 | 209228 | 212419 | 214019 | 214524 | 221319 | 233326 | 239136 | 246133 |
| 249947 | 251833 | 257845 | 263436 | 283543 | 318637 | 320826 | 330324 | 346137 | 352738 |
| 360030 | 365343 | 380036 | 381038 | 385046 | 389357 | 393247 | 395756 | 397760 | 401422 |
| 406836 | 412528 | 429040 | 436845 | 463040 | 467351 | 525541 | 541943 | 547046 | 562345 |
| 572045 | 573249 | 581450 | 601127 | 617748 | 627549 | 638049 | 644246 | 654552 | 671855 |
| 680957 | 690152 | 706040 | 724446 | 725751 | 732243 | 751550 | 757764 | 765864 | 770352 |
| 771354 | 782561 | 783462 | 789373 | 814649 | 818859 | 825048 | 826353 | 833249 | 859671 |
| 861456 | 871762 | 872764 | 878776 | 878877 | 898176 | 899885 | 901139 | 901442 | 905147 |
| 910241 | 926862 | 927359 | 942658 | 943862 | 951457 | 958976 | 966975 | 971160 |  |

**Table S2.** List of parcels whose eigenvector centralities were significant, corresponding to Figs. 4A and B. All p-values are below P < 0.0001.

| MOVIE > RET, 70 parcels | | | | | | | |
| --- | --- | --- | --- | --- | --- | --- | --- |
| Parcel | t-value | Parcel | t-value | Parcel | t-value | Parcel | t-value |
| R V1 | 28.5 | R 47m | 14.1 | R VVC | 14.1 | L LIPv | 4.7 |
| R MST | 6.2 | R ProS | 4.5 | R STSva | 4.5 | L ProS | 9.6 |
| R V6 | 4.4 | R PeEc | 27.9 | L V1 | 27.9 | L PeEc | 5.1 |
| R V2 | 33.3 | R STGa | 8.4 | L MST | 8.4 | L PHA1 | 16.6 |
| R V3 | 39.2 | R PHA1 | 32.6 | L V2 | 32.6 | L PHA3 | 20.3 |
| R V4 | 36.2 | R PHA3 | 39.6 | L V3 | 39.6 | L TF | 8.9 |
| R V8 | 33.9 | R STSda | 33.4 | L V4 | 33.4 | L TPOJ3 | 9.8 |
| R V3A | 29.3 | R TF | 29.8 | L V8 | 29.8 | L DVT | 7.3 |
| R V7 | 18.9 | R TPOJ3 | 25.1 | L V3A | 25.1 | L V6A | 12.8 |
| R FFC | 5.7 | R V6A | 19.2 | L V7 | 19.2 | L VMV1 | 16.7 |
| R V3B | 20.6 | R VMV1 | 4.7 | L FFC | 4.7 | L VMV3 | 22.0 |
| R LO1 | 20.9 | R VMV3 | 20.8 | L V3B | 20.8 | L PHA2 | 14.4 |
| R LO2 | 13.7 | R PHA2 | 21.0 | L LO1 | 21.0 | L V4t | 15.6 |
| R PIT | 8.3 | R V4t | 13.9 | L LO2 | 13.9 | L V3CD | 10.2 |
| R MT | 16.6 | R FST | 7.6 | L PIT | 7.6 | L LO3 | 22.3 |
| R POS1 | 10.6 | R V3CD | 17.4 | L MT | 17.4 | L VMV2 | 30.9 |
| R LIPv | 7.0 | R LO3 | 7.0 | L POS1 | 7.0 | L VVC | 12.7 |
| R VIP | 5.1 | R VMV2 | 14.1 |  |  |  |  |
| MOVIE > REST, 79 parcels | | | | | | | |
| Parcel | t-value | Parcel | t-value | Parcel | t-value | Parcel | t-value |
| R V1 | 12.1 | R IFJp | 11.4 | R VMV2 | 9.6 | L IFJp | 8.5 |
| R MST | 5.8 | R IFSp | 16.8 | R VVC | 13.3 | L IFSp | 9.0 |
| R V3 | 7.9 | R LIPd | 7.2 | R STSva | 4.4 | L LIPd | 4.5 |
| R V4 | 10.1 | R PeEc | 6.1 | L V1 | 13.0 | L PeEc | 5.8 |
| R V8 | 11.1 | R STGa | 4.6 | L MST | 4.5 | L PHA1 | 14.8 |
| R PEF | 6.0 | R PHA1 | 17.4 | L V3 | 6.9 | L PHA3 | 24.1 |
| R V7 | 5.5 | R PHA3 | 24.1 | L V4 | 11.4 | L TF | 10.7 |
| R IPS1 | 8.5 | R STSda | 12.9 | L V8 | 12.4 | L TE2p | 12.6 |
| R V3B | 9.3 | R TF | 12.5 | L PEF | 5.1 | L PH | 9.6 |
| R LO1 | 10.3 | R TE2p | 13.7 | L V7 | 4.3 | L TPOJ3 | 11.1 |
| R LO2 | 7.2 | R PH | 8.7 | L IPS1 | 6.8 | L PGp | 13.6 |
| R MT | 10.9 | R TPOJ3 | 15.2 | L FFC | 4.6 | L IP0 | 13.7 |
| R STV | 7.5 | R PGp | 12.9 | L V3B | 7.8 | L VMV3 | 10.3 |
| R POS1 | 10.9 | R IP0 | 11.9 | L LO1 | 11.8 | L PHA2 | 13.1 |
| R LIPv | 7.5 | R VMV3 | 10.2 | L LO2 | 7.6 | L V4t | 6.2 |
| R 47m | 5.7 | R PHA2 | 16.4 | L MT | 7.6 | L V3CD | 9.1 |
| R 8C | 5.2 | R V4t | 5.8 | L POS1 | 6.1 | L LO3 | 6.6 |
| R 45 | 5.5 | R FST | 4.4 | L LIPv | 5.8 | L VMV2 | 14.1 |
| R 47l | 4.3 | R V3CD | 9.8 | L MIP | 5.0 | L VVC | 12.4 |
| R IFJa | 12.7 | R LO3 | 7.8 | L IFJa | 9.0 |  |  |

R, right; L, left; Parcel labels were taken from Glasser et al., 2016.

**Table S3.** List of parcels whose participation coefficients were significant, corresponding to Figs. 4C and D. All p-values are below P < 0.0001.

| MOVIE > RET, 64 parcels | | | | | | | |
| --- | --- | --- | --- | --- | --- | --- | --- |
| Parcel | t-value | Parcel | t-value | Parcel | t-value | Parcel | t-value |
| R MT | 5.4 | R PBelt | 8.5 | L 7m | 10.3 | L 10v | 4.9 |
| R A1 | 4.4 | R A5 | 4.5 | L POS1 | 26.1 | L 52 | 7.6 |
| R PSL | 8.4 | R PHA1 | 7.9 | L v23ab | 11.8 | L RI | 10.9 |
| R 7m | 5.4 | R STSda | 10.9 | L d23ab | 7.0 | L PBelt | 8.2 |
| R POS1 | 12.5 | R TGd | 6.3 | L 31pv | 11.9 | L PHA1 | 9.1 |
| R v23ab | 10.6 | R TE1a | 4.9 | L VIP | 7.9 | L TGd | 5.0 |
| R 7PL | 4.7 | R TF | 5.9 | L a24 | 5.7 | L TE1a | 8.6 |
| R VIP | 9.9 | R PGi | 8.2 | L 10r | 5.3 | L TF | 10.5 |
| R 10r | 6.0 | R V4t | 5.7 | L 47m | 5.3 | L PGi | 9.4 |
| R 47m | 8.2 | R FST | 5.4 | L 8Ad | 7.8 | L PGs | 6.6 |
| R 8Ad | 4.4 | R 31pd | 4.8 | L 9m | 7.9 | L PHA2 | 6.5 |
| R 9m | 6.6 | R LBelt | 7.7 | L 8BL | 6.3 | L 31pd | 14.2 |
| R 10v | 5.1 | R A4 | 7.4 | L 9p | 9.3 | L MBelt | 5.7 |
| R 52 | 5.7 | R STSva | 8.5 | L 10d | 9.0 | L LBelt | 7.9 |
| R RI | 17.5 | L A1 | 4.8 | L IFSp | 5.3 | L A4 | 4.4 |
| R STGa | 5.1 | L PCV | 4.7 | L 9a | 5.9 | L STSva | 6.6 |
| MOVIE > REST, 48 parcels | | | | | | | |
| Parcel | t-value | Parcel | t-value | Parcel | t-value | Parcel | t-value |
| R PSL | 10.6 | R 47l | 6.7 | L 7m | 12.3 | L IFSp | 7.7 |
| R 7Pm | 4.6 | R IFSa | 5.9 | L POS1 | 21.4 | L IFSa | 4.9 |
| R 7m | 12.6 | R 10v | 5.3 | L v23ab | 10.6 | L 9a | 7.3 |
| R POS1 | 13.1 | R RI | 6.7 | L d23ab | 7.6 | L RI | 4.4 |
| R v23ab | 12.6 | R STSda | 7.9 | L 31pv | 13.5 | L STSvp | 5.9 |
| R 31pv | 7.9 | R STSvp | 6.2 | L 10r | 4.7 | L TE1a | 8.2 |
| R 10r | 5.9 | R TGd | 4.9 | L 8Av | 8.2 | L TF | 9.0 |
| R 47m | 7.7 | R TE1a | 6.9 | L 8Ad | 8.4 | L PGi | 11.4 |
| R 8Ad | 11.4 | R TF | 5.6 | L 9m | 8.9 | L PGs | 9.1 |
| R 9m | 11.8 | R PGi | 18.5 | L 8BL | 8.7 | L 31pd | 15.2 |
| R 8BL | 7.6 | R 31pd | 11.6 | L 9p | 10.0 | L 31a | 4.6 |
| R 10d | 6.1 | R STSva | 8.8 | L 10d | 8.1 | L STSva | 5.9 |

R, right; L, left; Parcel labels were taken from Glasser et al., 2016.

**Table S4.** List of parcels whose activation were significantly correlated with saliency, corresponding to Fig. 5A. All p-values are below P < 0.0001.

| Activation correlated with visual saliency, 199 parcels | | | | | | | | | | |
| --- | --- | --- | --- | --- | --- | --- | --- | --- | --- | --- |
| Parcel | t-val | Parcel | t-val | Parcel | t-val | Parcel | t-val | Parcel | t-val | |
| R V1 | 27.9 | R 8Ad | 8.3 | R V6A | 17.0 | L POS1 | 8.9 | L EC | 9.3 | |
| R MST | 54.3 | R 9m | 9.5 | R VMV1 | 10.5 | L 5m | 7.8 | L PeEc | 21.0 | |
| R V2 | 32.2 | R 8BL | 7.3 | R VMV3 | 29.7 | L 5mv | 5.7 | L STGa | 17.0 | |
| R V3 | 34.7 | R 45 | 6.5 | R PHA2 | 21.4 | L 5L | 5.0 | L PBelt | 41.2 | |
| R V4 | 38.6 | R 47l | 7.4 | R V4t | 53.2 | L 24dd | 6.9 | L A5 | 35.5 | |
| R V8 | 40.9 | R IFJa | 15.6 | R FST | 38.9 | L 7AL | 5.5 | L PHA1 | 21.5 | |
| R FEF | 14.0 | R IFJp | 10.9 | R V3CD | 32.2 | L 7Am | 5.9 | L PHA3 | 22.5 | |
| R PEF | 10.2 | R IFSp | 14.2 | R LO3 | 38.2 | L 7PL | 6.1 | L STSda | 16.9 | |
| R 55b | 13.8 | R IFSa | 5.6 | R VMV2 | 30.5 | L 7PC | 15.7 | L STSdp | 12.6 | |
| R V3A | 23.9 | R 10v | 15.9 | R VVC | 30.4 | L LIPv | 22.5 | L TGd | 9.9 | |
| R V7 | 29.0 | R LIPd | 7.4 | R 25 | 5.7 | L VIP | 16.5 | L TE1a | 11.8 | |
| R IPS1 | 24.4 | R 6a | 8.3 | R s32 | 9.5 | L MIP | 9.0 | L TF | 20.6 | |
| R FFC | 43.4 | R RI | 13.7 | R TGv | 12.4 | L 1 | 5.8 | L TE2p | 24.3 | |
| R V3B | 28.4 | R TA2 | 31.7 | R MBelt | 30.1 | L 2 | 8.4 | L PHT | 7.5 | |
| R LO1 | 38.2 | R Pir | 9.4 | R LBelt | 32.8 | L 6d | 5.8 | L PH | 20.3 | |
| R LO2 | 38.0 | R PFt | 10.0 | R A4 | 41.6 | L p24pr | 6.8 | L TPOJ1 | 19.3 | |
| R PIT | 30.2 | R AIP | 7.1 | R STSva | 11.5 | L 33pr | 7.6 | L TPOJ2 | 25.5 | |
| R MT | 51.6 | R EC | 8.3 | L V1 | 26.0 | L 10r | 9.9 | L TPOJ3 | 31.0 | |
| R A1 | 28.4 | R H | 7.1 | L MST | 45.6 | L 47m | 14.1 | L DVT | 13.8 | |
| R PCV | 13.0 | R PeEc | 21.6 | L V2 | 29.5 | L 8Ad | 11.0 | L PGp | 20.9 | |
| R STV | 16.9 | R STGa | 15.0 | L V3 | 31.3 | L 9m | 8.1 | L IP0 | 20.1 | |
| R 7m | 6.1 | R PBelt | 35.4 | L V4 | 33.3 | L 8BL | 6.5 | L PGi | 9.1 | |
| R POS1 | 9.8 | R A5 | 40.1 | L V8 | 36.9 | L 45 | 5.2 | L V6A | 13.4 | |
| R v23ab | 4.7 | R PHA1 | 23.5 | L FEF | 13.0 | L 47l | 4.5 | L VMV1 | 10.9 | |
| R 5m | 6.1 | R PHA3 | 24.0 | L PEF | 10.2 | L IFJa | 15.8 | L VMV3 | 30.8 | |
| R 5mv | 7.6 | R STSda | 18.0 | L 55b | 9.9 | L IFJp | 11.4 | L PHA2 | 18.3 | |
| R 24dd | 5.6 | R STSdp | 13.6 | L V3A | 20.4 | L IFSp | 12.2 | L V4t | 44.4 | |
| R 7AL | 5.3 | R TGd | 11.2 | L V7 | 23.0 | L IFSa | 7.8 | L FST | 32.8 | |
| R 7Am | 6.2 | R TE1a | 14.7 | L IPS1 | 24.5 | L 10v | 14.8 | L V3CD | 28.9 | |
| R 7PC | 17.2 | R TF | 26.6 | L FFC | 37.7 | L LIPd | 6.0 | L LO3 | 35.0 | |
| R LIPv | 24.1 | R TE2p | 31.1 | L V3B | 25.8 | L 6a | 9.9 | L VMV2 | 30.5 | |
| R VIP | 20.0 | R PHT | 7.7 | L LO1 | 34.5 | L OP1 | 4.7 | L VVC | 29.1 | |
| R MIP | 5.5 | R PH | 23.9 | L LO2 | 29.4 | L 52 | 5.6 | L 25 | | 7.2 |
| R 1 | 6.8 | R TPOJ1 | 21.7 | L PIT | 22.3 | L RI | 21.8 | L s32 | | 11.1 |
| R 2 | 11.1 | R TPOJ2 | 26.2 | L MT | 47.3 | L PFcm | 4.4 | L MBelt | | 33.7 |
| R 6d | 8.5 | R TPOJ3 | 35.5 | L A1 | 33.1 | L TA2 | 30.9 | L LBelt | | 35.4 |
| R p24pr | 5.8 | R DVT | 14.7 | L PSL | 6.7 | L Pir | 9.1 | L A4 | | 43.5 |
| R 33pr | 5.6 | R PGp | 21.3 | L PCV | 14.6 | L FOP2 | 5.3 | L STSva | | 11.6 |
| R 10r | 8.4 | R IP0 | 18.7 | L STV | 20.7 | L PFt | 9.4 | L PI | | 8.4 |
| R 47m | 14.1 | R PGi | 6.9 | L 7m | 5.5 | L AIP | 6.4 |  | |  |

R, right; L, left; Parcel labels were taken from Glasser et al., 2016.

**Table S5.** List of parcels that the time-varying centralities were significantly correlated with the visual saliency, corresponding to Fig. 6.

| tEC > tPC, 38 parcels | | | | | | | |
| --- | --- | --- | --- | --- | --- | --- | --- |
| Parcel | t-value | Parcel | t-value | Parcel | t-value | Parcel | t-value |
| R 7Pm | 5.0 | R 6a | 11.3 | L 7AL | 5.0 | L 6a | 9.4 |
| R 7AL | 4.4 | R PFt | 8.6 | L 7Am | 5.3 | L PFt | 11.0 |
| R 7Am | 6.9 | R AIP | 13.6 | L 7PL | 5.9 | L AIP | 16.3 |
| R 7PL | 5.6 | R PHT | 15.1 | L 7PC | 9.6 | L PHT | 8.8 |
| R 7PC | 9.4 | R PGp | 5.6 | L VIP | 6.4 | L PH | 6.4 |
| R VIP | 5.4 | R FST | 4.4 | L MIP | 9.2 | L PGp | 4.4 |
| R MIP | 8.6 | R p24 | 4.9 | L p32 | 4.4 | L PFm | 4.3 |
| R 6r | 7.8 | L RSC | 4.5 | L 6r | 5.3 | L PGs | 4.6 |
| R IFSa | 8.4 | L POS2 | 4.7 | L LIPd | 4.8 | L FST | 8.3 |
| R LIPd | 5.0 | L 23d | 5.2 |  |  |  |  |
| tPC > tEC, 89 parcels | | | | | | | |
| Parcel | t-value | Parcel | t-value | Parcel | t-value | Parcel | t-value |
| R V1 | 13.6 | R 43 | 5.8 | R LBelt | 16.9 | L IFSp | 6.1 |
| R MST | 4.9 | R 52 | 6.1 | R A4 | 11.7 | L 43 | 4.5 |
| R V2 | 17.2 | R RI | 7.0 | R PI | 4.9 | L 52 | 7.3 |
| R V3 | 14.7 | R TA2 | 13.7 | L V1 | 11.2 | L RI | 9.8 |
| R V4 | 10.4 | R FOP4 | 5.5 | L V2 | 13.2 | L TA2 | 11.9 |
| R V8 | 10.8 | R MI | 6.0 | L V3 | 11.4 | L FOP4 | 5.2 |
| R 3b | 4.7 | R FOP1 | 4.5 | L V4 | 9.5 | L MI | 5.8 |
| R 55b | 6.5 | R FOP3 | 6.0 | L V8 | 10.9 | L FOP3 | 6.0 |
| R V3A | 11.5 | R PBelt | 14.4 | L 4 | 4.5 | L ProS | 4.8 |
| R FFC | 12.4 | R STSda | 7.3 | L V3A | 8.2 | L PeEc | 4.6 |
| R V3B | 7.5 | R STSdp | 5.4 | L FFC | 10.6 | L PBelt | 14.8 |
| R LO1 | 6.3 | R TGd | 5.3 | L V3B | 7.1 | L TF | 6.1 |
| R LO2 | 9.6 | R TF | 5.7 | L LO1 | 6.7 | L TPOJ1 | 9.3 |
| R PIT | 10.6 | R TPOJ1 | 10.6 | L LO2 | 8.0 | L VMV1 | 7.1 |
| R MT | 6.6 | R VMV1 | 8.3 | L PIT | 8.7 | L VMV3 | 7.1 |
| R A1 | 13.8 | R VMV3 | 7.5 | L MT | 5.6 | L V4t | 6.5 |
| R SFL | 5.2 | R V4t | 8.0 | L A1 | 14.3 | L VMV2 | 7.1 |
| R STV | 5.7 | R V3CD | 5.4 | L STV | 10.0 | L VVC | 6.5 |
| R a24pr | 5.2 | R VMV2 | 7.4 | L a24pr | 5.1 | L MBelt | 14.4 |
| R p32pr | 4.9 | R VVC | 5.9 | L p32pr | 4.5 | L LBelt | 17.1 |
| R 47m | 4.9 | R TGv | 6.0 | L 47m | 4.6 | L A4 | 8.6 |
| R 45 | 5.8 | R MBelt | 16.8 | L IFJa | 7.0 | L PI | 6.2 |
| R IFJa | 8.6 |  |  |  |  |  |  |

R, right; L, left; Parcel labels were taken from Glasser et al., 2016.

**Table S6.** List of parcels whose eigenvector centralities were significant, corresponding to Figs. S3. All p-values are below P < 0.0001.

| RET > MOVIE, 120 parcels | | | | | | | |
| --- | --- | --- | --- | --- | --- | --- | --- |
| Parcel | t-value | Parcel | t-value | Parcel | t-value | Parcel | t-value |
| R FEF | 8.0 | R 46 | 26.9 | R p47r | 12.0 | L p9-46v | 21.3 |
| R PEF | 6.2 | R a9-46v | 12.6 | R TE1m | 8.0 | L 46 | 18.1 |
| R 55b | 9.2 | R 9-46d | 17.0 | R a32pr | 13.6 | L a9-46v | 13.2 |
| R POS2 | 12.2 | R a10p | 8.4 | L FEF | 10.6 | L 9-46d | 13.5 |
| R PSL | 15.7 | R 10pp | 5.0 | L PEF | 7.4 | L a10p | 6.0 |
| R SFL | 4.6 | R 11l | 10.4 | L 55b | 5.2 | L 11l | 7.6 |
| R 7Pm | 22.8 | R 13l | 5.3 | L POS2 | 7.5 | L LIPd | 19.2 |
| R 23d | 6.0 | R LIPd | 13.5 | L PSL | 4.6 | L 6a | 22.2 |
| R d23ab | 5.3 | R 6a | 21.5 | L 7Pm | 13.0 | L i6-8 | 12.1 |
| R 23c | 16.7 | R i6-8 | 17.9 | L 23c | 9.0 | L s6-8 | 7.5 |
| R SCEF | 13.2 | R s6-8 | 9.7 | L 7AL | 4.8 | L FOP4 | 18.0 |
| R 6ma | 22.9 | R FOP4 | 19.3 | L SCEF | 11.8 | L MI | 8.1 |
| R 7Am | 19.5 | R MI | 10.3 | L 6ma | 16.8 | L AVI | 13.1 |
| R 7PL | 19.1 | R AVI | 15.9 | L 7Am | 17.9 | L FOP1 | 5.6 |
| R 7PC | 4.9 | R FOP1 | 4.9 | L 7PL | 21.5 | L PFt | 24.2 |
| R MIP | 14.6 | R PFt | 22.2 | L 7PC | 6.4 | L AIP | 31.0 |
| R 6v | 9.7 | R AIP | 26.4 | L MIP | 15.5 | L TE1p | 10.3 |
| R a24pr | 5.1 | R STSvp | 7.5 | L 6v | 9.2 | L PHT | 18.1 |
| R p32pr | 12.7 | R TE1p | 15.6 | L a24pr | 5.2 | L TPOJ2 | 5.5 |
| R d32 | 9.9 | R PHT | 16.2 | L p32pr | 10.7 | L IP2 | 24.3 |
| R 8BM | 17.8 | R TPOJ2 | 4.4 | L d32 | 5.3 | L IP1 | 19.5 |
| R 8Av | 11.1 | R IP2 | 25.8 | L 8BM | 12.8 | L IP0 | 4.9 |
| R 9p | 5.0 | R IP1 | 19.8 | L 8Av | 4.5 | L PFop | 13.3 |
| R 8C | 11.8 | R PFop | 16.3 | L 8C | 9.9 | L PF | 23.2 |
| R 44 | 16.5 | R PF | 27.0 | L 44 | 7.1 | L PFm | 14.0 |
| R a47r | 12.0 | R PFm | 16.3 | L a47r | 5.3 | L FOP5 | 15.7 |
| R 6r | 31.4 | R PGs | 10.3 | L 6r | 26.3 | L p10p | 6.4 |
| R IFJp | 6.3 | R 31a | 4.5 | L IFJa | 4.7 | L p47r | 12.0 |
| RET > REST, 102 parcels | | | | | | | |
| Parcel | t-value | Parcel | t-value | Parcel | t-value | Parcel | t-value |
| R FEF | 4.6 | R p9-46v | 19.3 | R PFm | 15.4 | L IFSa | 12.5 |
| R PEF | 15.9 | R 46 | 13.8 | R PGs | 7.5 | L p9-46v | 19.5 |
| R 55b | 7.4 | R a9-46v | 10.2 | R 31a | 5.7 | L 46 | 5.1 |
| R POS2 | 10.5 | R 9-46d | 9.0 | R FOP5 | 8.5 | L a9-46v | 11.2 |
| R IPS1 | 4.9 | R a10p | 7.5 | R p10p | 7.4 | L 9-46d | 5.2 |
| R PSL | 5.7 | R 11l | 9.3 | R p47r | 10.9 | L a10p | 6.1 |
| R PCV | 7.0 | R LIPd | 24.2 | R TE1m | 7.7 | L LIPd | 25.0 |
| R STV | 9.7 | R 6a | 20.4 | R a32pr | 8.3 | L 6a | 20.9 |
| R 7Pm | 21.4 | R i6-8 | 16.3 | L FEF | 5.2 | L i6-8 | 9.8 |
| R 6ma | 8.0 | R s6-8 | 8.7 | L PEF | 14.7 | L s6-8 | 4.6 |
| R 7Am | 12.5 | R AVI | 15.3 | L 55b | 5.2 | L AVI | 12.7 |
| R 7PL | 20.3 | R PFt | 15.3 | L POS2 | 5.6 | L PFt | 18.1 |
| R MIP | 19.5 | R AIP | 20.2 | L 7Pm | 11.8 | L AIP | 27.2 |
| R d32 | 8.5 | R PHA3 | 8.2 | L 6ma | 6.7 | L TE1p | 7.7 |
| R 8BM | 17.3 | R STSvp | 8.9 | L 7Am | 11.4 | L TE2p | 13.2 |
| R 8Av | 8.7 | R TE1p | 15.6 | L 7PL | 21.6 | L PHT | 14.9 |
| R 9p | 5.4 | R TF | 4.5 | L MIP | 20.3 | L PH | 11.3 |
| R 8C | 14.7 | R TE2p | 12.0 | L 8BM | 11.3 | L PGp | 14.0 |
| R 44 | 13.6 | R PHT | 16.0 | L 8C | 9.0 | L IP2 | 20.8 |
| R 47l | 5.7 | R PH | 5.7 | L 44 | 5.9 | L IP1 | 18.2 |
| R a47r | 11.3 | R TPOJ3 | 9.1 | L a47r | 4.7 | L IP0 | 17.9 |
| R 6r | 14.9 | R PGp | 15.5 | L 6r | 16.1 | L PF | 7.9 |
| R IFJa | 15.3 | R IP2 | 22.6 | L IFJa | 12.4 | L PFm | 9.3 |
| R IFJp | 20.9 | R IP1 | 18.7 | L IFJp | 22.6 | L FOP5 | 5.3 |
| R IFSp | 19.9 | R IP0 | 14.2 | L IFSp | 9.5 | L p47r | 10.4 |
| R IFSa | 11.2 | R PF | 10.9 | R PFm | 15.4 | L IFSa | 12.5 |
| REST > MOVIE, 105 parcels | | | | | | | |
| Parcel | t-value | Parcel | t-value | Parcel | t-value | Parcel | t-value |
| R V6 | 21.5 | R 9-46d | 7.3 | L V6 | 22.6 | L 9-46d | 6.7 |
| R 4 | 10.9 | R 43 | 11.2 | L 4 | 10.9 | L 11l | 4.8 |
| R 3b | 9.7 | R OP4 | 11.2 | L 3b | 10.0 | L 43 | 10.7 |
| R PSL | 7.7 | R OP1 | 6.1 | L FEF | 4.9 | L OP4 | 11.3 |
| R 5m | 8.9 | R OP2-3 | 6.3 | L 5m | 8.1 | L OP1 | 7.0 |
| R 5mv | 15.5 | R RI | 9.3 | L 5mv | 11.8 | L OP2-3 | 7.6 |
| R 23c | 11.5 | R PFcm | 8.8 | L 23c | 10.7 | L 52 | 5.0 |
| R 5L | 8.1 | R PoI2 | 7.0 | L 5L | 7.3 | L RI | 8.9 |
| R 24dd | 9.2 | R FOP4 | 10.9 | L 24dd | 8.3 | L PFcm | 9.0 |
| R 24dv | 8.6 | R MI | 10.6 | L 24dv | 8.0 | L PoI2 | 6.2 |
| R 7AL | 7.3 | R FOP1 | 9.5 | L 7AL | 8.5 | L FOP4 | 10.4 |
| R SCEF | 11.9 | R FOP3 | 8.9 | L SCEF | 10.0 | L MI | 9.3 |
| R 6ma | 10.7 | R FOP2 | 5.0 | L 6ma | 7.7 | L FOP1 | 9.6 |
| R 7Am | 7.3 | R PFt | 8.4 | L 7Am | 6.5 | L FOP3 | 8.3 |
| R 7PC | 8.4 | R AIP | 5.4 | L 7PC | 7.8 | L FOP2 | 4.7 |
| R 1 | 11.9 | R PBelt | 4.5 | L 1 | 11.1 | L PFt | 6.5 |
| R 2 | 11.2 | R A5 | 4.3 | L 2 | 11.0 | L PBelt | 5.5 |
| R 3a | 10.3 | R IP2 | 6.9 | L 3a | 10.4 | L A5 | 4.6 |
| R 6d | 6.1 | R PFop | 11.5 | L 6d | 5.6 | L IP2 | 5.6 |
| R 6mp | 8.7 | R PF | 10.6 | L 6mp | 8.3 | L PFop | 9.6 |
| R 6v | 10.1 | R VMV1 | 4.6 | L 6v | 8.1 | L PF | 9.9 |
| R p24pr | 8.9 | R PoI1 | 8.3 | L p24pr | 4.9 | L V6A | 4.4 |
| R a24pr | 10.4 | R Ig | 7.0 | L a24pr | 10.2 | L PoI1 | 7.5 |
| R p32pr | 12.4 | R FOP5 | 8.5 | L p32pr | 11.3 | L Ig | 5.3 |
| R 6r | 9.9 | R A4 | 5.8 | L 6r | 8.2 | L FOP5 | 7.8 |
| R 46 | 9.4 | R a32pr | 6.1 | L 46 | 9.6 | L A4 | 7.9 |
| R a9-46v | 4.5 |  |  |  |  |  |  |
| REST > RET, 96 parcels | | | | | | | |
| Parcel | t-value | Parcel | t-value | Parcel | t-value | Parcel | t-value |
| R V1 | 13.9 | R a24pr | 7.6 | L V6 | 25.5 | L a24pr | 7.6 |
| R V6 | 23.6 | R 43 | 10.6 | L V2 | 25.8 | L 43 | 10.4 |
| R V2 | 26.1 | R OP4 | 10.1 | L V3 | 24.6 | L OP4 | 8.4 |
| R V3 | 21.3 | R OP1 | 7.2 | L V4 | 15.2 | L OP1 | 7.3 |
| R V4 | 16.9 | R OP2-3 | 6.8 | L V8 | 10.6 | L OP2-3 | 7.8 |
| R V8 | 13.7 | R 52 | 4.8 | L 4 | 10.0 | L 52 | 5.8 |
| R 4 | 10.9 | R RI | 8.7 | L 3b | 8.3 | L RI | 8.2 |
| R 3b | 8.5 | R PFcm | 9.3 | L V3A | 28.2 | L PFcm | 8.6 |
| R V3A | 28.3 | R PoI2 | 10.2 | L V7 | 12.2 | L PoI2 | 8.9 |
| R V7 | 9.6 | R FOP1 | 7.1 | L V3B | 9.9 | L FOP1 | 6.4 |
| R V3B | 9.2 | R FOP3 | 9.0 | L LO1 | 6.8 | L FOP3 | 7.8 |
| R LO1 | 7.2 | R FOP2 | 6.6 | L LO2 | 4.8 | L FOP2 | 4.6 |
| R LO2 | 4.8 | R ProS | 11.7 | L A1 | 4.8 | L ProS | 10.9 |
| R 5m | 9.2 | R PBelt | 6.1 | L 5m | 8.9 | L PBelt | 6.3 |
| R 5mv | 13.4 | R V6A | 14.8 | L 5mv | 10.5 | L DVT | 6.0 |
| R 5L | 8.9 | R VMV1 | 19.7 | L 5L | 8.4 | L V6A | 18.0 |
| R 24dd | 8.2 | R VMV3 | 5.3 | L 24dd | 6.8 | L VMV1 | 18.8 |
| R 24dv | 8.1 | R LO3 | 12.5 | L 24dv | 8.1 | L VMV3 | 6.8 |
| R 7AL | 7.8 | R VMV2 | 11.6 | L 7AL | 4.8 | L V4t | 5.7 |
| R 1 | 10.3 | R PoI1 | 9.4 | L 1 | 9.0 | L LO3 | 13.4 |
| R 2 | 10.2 | R Ig | 8.5 | L 2 | 10.2 | L VMV2 | 9.4 |
| R 3a | 10.0 | R LBelt | 6.7 | L 3a | 9.8 | L PoI1 | 9.5 |
| R 6mp | 7.8 | R A4 | 7.1 | L 6mp | 6.9 | L Ig | 6.8 |
| R p24pr | 6.8 | L V1 | 12.0 | L p24pr | 4.5 | L A4 | 6.9 |

R, right; L, left; Parcel labels were taken from Glasser et al., 2016.

**Table S7.** List of parcels whose participation coefficients were significant, corresponding to Figs. S4. All p-values are below P < 0.0001.

| RET > MOVIE, 96 parcels | | | | | | | |
| --- | --- | --- | --- | --- | --- | --- | --- |
| Parcel | t-value | Parcel | t-value | Parcel | t-value | Parcel | t-value |
| R V1 | 11.0 | R a9-46v | 6.1 | L V1 | 10.4 | L a9-46v | 7.7 |
| R V2 | 7.7 | R LIPd | 6.7 | L V2 | 7.8 | L 6a | 5.5 |
| R 4 | 4.7 | R i6-8 | 6.9 | L V3 | 5.3 | L i6-8 | 8.3 |
| R 3b | 5.1 | R FOP4 | 6.0 | L 4 | 6.5 | L s6-8 | 5.4 |
| R FEF | 9.6 | R FOP1 | 9.5 | L 3b | 7.5 | L FOP4 | 5.2 |
| R PEF | 10.1 | R TE1p | 7.2 | L FEF | 7.5 | L AVI | 4.5 |
| R V3A | 8.1 | R TE2p | 6.3 | L PEF | 10.7 | L FOP1 | 9.1 |
| R RSC | 7.8 | R PH | 5.0 | L 55b | 8.1 | L PFt | 6.4 |
| R POS2 | 17.2 | R DVT | 13.1 | L V3A | 8.8 | L PHA3 | 4.8 |
| R V3B | 4.9 | R PGp | 6.0 | L RSC | 6.2 | L TE1p | 8.7 |
| R 23d | 6.9 | R IP2 | 8.6 | L POS2 | 12.9 | L PH | 10.3 |
| R 23c | 4.6 | R IP1 | 10.2 | L V3B | 5.9 | L DVT | 9.8 |
| R 7AL | 4.4 | R IP0 | 13.6 | L PSL | 5.8 | L PGp | 7.3 |
| R SCEF | 13.3 | R PFop | 12.2 | L SFL | 4.6 | L IP2 | 14.9 |
| R 6ma | 7.8 | R PF | 12.1 | L 7AL | 4.7 | L IP1 | 11.9 |
| R 6v | 12.6 | R PFm | 12.0 | L SCEF | 14.3 | L IP0 | 11.6 |
| R p32pr | 9.2 | R PGs | 7.4 | L 6ma | 5.1 | L PFop | 9.0 |
| R d32 | 10.2 | R VMV3 | 4.6 | L 3a | 4.6 | L PF | 7.5 |
| R 8BM | 7.7 | R V3CD | 4.7 | L 6v | 9.6 | L PFm | 10.0 |
| R 8Av | 5.3 | R VMV2 | 4.6 | L p32pr | 7.8 | L VMV3 | 4.6 |
| R a47r | 8.4 | R VVC | 9.6 | L 8BM | 9.4 | L V3CD | 6.5 |
| R 6r | 8.8 | R p10p | 5.5 | L 8C | 5.9 | L VVC | 12.4 |
| R IFJp | 5.0 | R p47r | 7.6 | L 6r | 9.9 | L p10p | 5.3 |
| R p9-46v | 9.8 | R TE1m | 6.7 | L p9-46v | 9.8 | L p47r | 8.5 |
| RET > REST, 60 parcels | | | | | | | |
| Parcel | t-value | Parcel | t-value | Parcel | t-value | Parcel | t-value |
| R PEF | 5.1 | R 6r | 6.6 | R PGi | 7.8 | L 46 | 5.4 |
| R RSC | 4.7 | R IFJp | 5.9 | R PGs | 9.4 | L 6a | 4.6 |
| R POS2 | 7.2 | R IFSp | 5.1 | R 31pd | 5.8 | L i6-8 | 6.0 |
| R 7m | 6.5 | R IFSa | 6.2 | R 31a | 6.4 | L s6-8 | 4.5 |
| R 23d | 7.4 | R p9-46v | 5.9 | R p10p | 4.6 | L FOP4 | 5.1 |
| R 31pv | 5.6 | R 46 | 5.0 | L RSC | 4.8 | L AVI | 4.8 |
| R 6ma | 7.5 | R i6-8 | 5.4 | L POS2 | 7.2 | L TE1p | 8.2 |
| R 8BM | 4.4 | R s6-8 | 5.8 | L 8BM | 6.8 | L PHT | 5.8 |
| R 8Av | 8.2 | R FOP4 | 6.3 | L 8Av | 5.6 | L IP2 | 5.2 |
| R 8Ad | 7.2 | R STSvp | 9.2 | L 8C | 7.5 | L IP1 | 8.1 |
| R 9m | 4.9 | R TE1p | 8.1 | L 44 | 5.4 | L PF | 8.0 |
| R 8BL | 7.7 | R PHT | 4.5 | L a47r | 5.5 | L PFm | 7.9 |
| R 8C | 4.9 | R IP1 | 6.5 | L 6r | 6.8 | L VVC | 8.1 |
| R 47l | 6.1 | R PF | 9.9 | L IFJp | 4.5 | L p47r | 4.6 |
| R a47r | 4.8 | R PFm | 4.3 | L p9-46v | 6.5 | L TE1m | 4.3 |
| REST > MOVIE, 107 parcels | | | | | | | |
| Parcel | t-value | Parcel | t-value | Parcel | t-value | Parcel | t-value |
| R V1 | 12.6 | R d32 | 5.8 | R VMV2 | 4.7 | L 6v | 8.5 |
| R V6 | 5.9 | R a9-46v | 7.0 | R VVC | 5.4 | L p24pr | 4.4 |
| R V2 | 16.3 | R 11l | 5.7 | R PoI1 | 4.6 | L a24pr | 7.0 |
| R V3 | 8.2 | R LIPd | 4.5 | R Ig | 6.8 | L p32pr | 11.5 |
| R V4 | 4.4 | R 43 | 14.0 | R p47r | 5.1 | L a9-46v | 6.8 |
| R 4 | 14.8 | R OP4 | 8.9 | L V1 | 10.6 | L 43 | 13.5 |
| R 3b | 12.8 | R OP1 | 4.6 | L V6 | 7.4 | L OP4 | 8.6 |
| R FEF | 7.8 | R OP2-3 | 5.4 | L V2 | 14.4 | L OP2-3 | 7.3 |
| R PEF | 4.4 | R PFcm | 6.8 | L V3 | 9.5 | L PoI2 | 8.5 |
| R V3A | 10.7 | R PoI2 | 8.5 | L 4 | 15.3 | L FOP1 | 10.9 |
| R POS2 | 8.0 | R FOP1 | 10.6 | L 3b | 13.2 | L FOP3 | 5.5 |
| R 5m | 10.5 | R FOP3 | 5.3 | L FEF | 7.4 | L FOP2 | 4.4 |
| R 5mv | 8.3 | R PFt | 6.3 | L PEF | 6.3 | L ProS | 6.3 |
| R 5L | 9.5 | R ProS | 6.7 | L V3A | 10.0 | L A5 | 6.2 |
| R 24dd | 8.1 | R PHA3 | 8.9 | L V3B | 4.8 | L PHA3 | 13.1 |
| R 24dv | 10.9 | R TE2p | 6.1 | L 5m | 9.2 | L STSdp | 5.6 |
| R 7AL | 8.0 | R PH | 10.9 | L 5mv | 8.0 | L PH | 12.1 |
| R SCEF | 14.2 | R TPOJ3 | 7.8 | L 5L | 7.4 | L TPOJ3 | 4.6 |
| R LIPv | 6.9 | R DVT | 14.5 | L 24dd | 8.6 | L DVT | 13.3 |
| R 1 | 13.7 | R PGp | 10.3 | L 24dv | 10.1 | L PGp | 8.9 |
| R 2 | 7.6 | R IP2 | 6.9 | L 7AL | 7.2 | L IP2 | 10.0 |
| R 3a | 14.1 | R IP0 | 14.0 | L SCEF | 14.7 | L IP0 | 12.5 |
| R 6mp | 9.4 | R PFop | 10.4 | L LIPv | 5.6 | L PFop | 7.8 |
| R 6v | 11.3 | R PFm | 6.5 | L 1 | 12.9 | L FST | 6.6 |
| R p24pr | 7.5 | R VMV1 | 5.6 | L 2 | 8.4 | L LO3 | 6.4 |
| R a24pr | 6.4 | R FST | 6.7 | L 3a | 14.6 | L PoI1 | 5.6 |
| R p32pr | 11.1 | R LO3 | 5.7 | L 6mp | 8.8 |  |  |
| REST > RET, 76 parcels | | | | | | | |
| Parcel | t-value | Parcel | t-value | Parcel | t-value | Parcel | t-value |
| R MST | 5.6 | R OP1 | 5.0 | R PoI1 | 5.5 | L a24pr | 4.9 |
| R V2 | 6.9 | R OP2-3 | 7.0 | R Ig | 8.4 | L 43 | 8.7 |
| R 4 | 12.2 | R 52 | 5.3 | R LBelt | 4.7 | L OP4 | 7.7 |
| R 3b | 8.8 | R RI | 11.5 | R A4 | 8.1 | L OP2-3 | 7.9 |
| R MT | 4.7 | R PFcm | 8.0 | L V2 | 5.4 | L 52 | 6.0 |
| R 5m | 10.8 | R PoI2 | 10.6 | L 4 | 11.4 | L RI | 7.8 |
| R 5mv | 5.5 | R FOP3 | 4.9 | L 3b | 7.8 | L PFcm | 5.6 |
| R 5L | 10.4 | R FOP2 | 5.7 | L POS1 | 5.6 | L PoI2 | 8.0 |
| R 24dd | 9.0 | R PFt | 4.4 | L 5m | 9.5 | L ProS | 8.3 |
| R 24dv | 10.5 | R ProS | 6.8 | L 5mv | 5.9 | L PBelt | 6.5 |
| R 7PC | 7.9 | R PBelt | 5.9 | L 5L | 11.3 | L A5 | 8.6 |
| R VIP | 10.1 | R A5 | 7.5 | L 24dd | 6.2 | L PHA1 | 10.6 |
| R 1 | 11.4 | R PHA1 | 8.5 | L 24dv | 9.2 | L PHA3 | 7.8 |
| R 2 | 8.6 | R PHA3 | 5.2 | L 7PC | 6.9 | L PHA2 | 5.6 |
| R 3a | 11.7 | R PH | 4.4 | L VIP | 9.6 | L FST | 8.7 |
| R 6mp | 8.0 | R PGp | 4.9 | L 1 | 10.2 | L LO3 | 4.5 |
| R p24pr | 4.5 | R V4t | 5.3 | L 2 | 7.6 | L PoI1 | 6.0 |
| R 43 | 9.9 | R FST | 9.7 | L 3a | 10.6 | L MBelt | 4.8 |
| R OP4 | 11.5 | R LO3 | 4.7 | L 6mp | 5.3 | L A4 | 8.6 |

R, right; L, left; Parcel labels were taken from Glasser et al., 2016.
